# Efficacy and Safety of Transcutaneous Electrical Acupoint Stimulation to Treat Muscle Spasticity following Brain Injury: A Double-Blinded, Multicenter, Randomized Controlled Trial

**DOI:** 10.1371/journal.pone.0116976

**Published:** 2015-02-02

**Authors:** Wenli Zhao, Chao Wang, Zhongzheng Li, Lei Chen, Jianbo Li, Weidong Cui, Shasha Ding, Qiang Xi, Fan Wang, Fei Jia, Shuhua Xiao, Yi Guo, Ye Zhao

**Affiliations:** 1 Department of Neurology, Tianjin Nankai Hospital, Tianjin, 300100, China; 2 Department of Acupuncture and Moxibustion, Tianjin University of Traditional Chinese Medicine, Tianjin, 300193, China; 3 Department of Acupuncture and Moxibustion, Tianjin Ninghe Hospital, Tianjin, 301500, China; 4 Department of Clinical Research, Tianjin Nankai Hospital and Tianjin Academy of Integrative Medicine, Tianjin, 300100, China; Griffith University, AUSTRALIA

## Abstract

**Objective:**

This study was aimed at evaluating the clinical efficacy and safety of transcutaneous electrical acupoint stimulation (TEAS) to treat muscle spasticity after brain injury (Chinese Clinical Trial Registry: ChiCTR-TRC-11001310).

**Methods:**

A total of 60 patients with muscle spasticity after brain injury were randomized to the following 3 groups: 100, 2, and 0 Hz (sham) TEAS. The acupoints Hegu (LI4)—Yuji (LU10) and Zusanli (ST36)—Chengshan (BL57) on the injured side were stimulated at 0, 2, or 100 Hz, 5 times per week for 4 weeks. The patients were followed up for 1 and 2 months after the treatments. The effects of the treatments on muscle spasticity at the wrist, thumb, the other 4 fingers, elbow, shoulder, knee, and ankle were evaluated by the Modified Ashworth Scale, and the effects on disability were assessed by the Disability Assessment Scale. The walking capability was evaluated by the Holden functional ambulation classification score. The overall performance was assessed by the Global Assessment Scale score and the improved Barthel Index. The safety of the treatments administered was also monitored.

**Results:**

The wrist spasticity was significantly reduced from baseline at weeks 2, 3, and 4 of treatment and at the 1- and 2-month follow-up visits in the 100 Hz group (*P* < 0.01). Compared with 2 Hz or sham TEAS, 100 Hz TEAS decreased wrist spasticity at weeks 2, 3, and 4 of treatment and 1 month after treatment (*P* < 0.001). The other endpoints were not affected by the treatments. No treatment-emergent adverse events were reported during treatments and follow-up visits.

**Conclusions:**

TEAS appears to be a safe and effective therapy to relieve muscle spasticity after brain injury, although large-scale studies are required to further verify the findings.

**Trial Registration:**

Chinese Clinical Trial Registry ChiCTR-TRC-11001310 http://www.chictr.org

## INTRODUCTION

Spasticity—characterized by exaggerated tendon jerks and clonus—is a common complication following brain or spinal cord damages, and adversely affects work and quality of life of patients [1–4]. The exact pathophysiology of spasticity remains unclear. Excessive activity of the alpha motor neuron (-MN) pool caused by the imbalance between inhibitory and excitatory effects is thought to contribute to spasticity after brain injury [5]. Brain and spinal cord injuries frequently damage the dorsal reticulospinal tract, which mediates inhibitory effects and, thus, consequently lead to the exaggeration of excitatory inputs from the medial reticulospinal and vestibulospinal tracts and, ultimately, spasticity [6].

Conventional therapies for spasticity including physical therapy, surgery, and pharmacotherapy often produce unsatisfactory outcomes. The outcomes of surgical intervention vary, depending upon the location and the severity of spasticity [7], whereas the effects of pharmacologic treatments are often short term for many patients and severe side effects occur sometimes [8–10]. Transcutaneous electrical nerve stimulation (TENS) has also been shown to improve clinical, electrophysiological, and functional variables in patients with spasticity with a similar efficacy as baclofen [11]. Similar to TENS, transcutaneous electrical acupoint stimulation (TEAS) is also an electrical stimulation method in which acupoints specifically associated with medical conditions are stimulated.

TEAS has, recently, been emerging as a popular therapeutic approach and has been widely tested in many medical conditions, such as pain, gastrointestinal disorder, inflammation, cancer, and Alzheimer [12–17]. Although controversial results have been reported, TEAS appears to be increasingly used in clinical practice now [18–20]. The mechanism of action of TEAS might be similar to that of electrical acupuncture (EA), in which acupoints are stimulated by electrical impulses given through needles. Studies on pain management using EA in animal models and patients suggest that EA blocks pain by activating a broad spectrum of bioactive chemicals such as opioids, serotonin, and norepinephrine to desensitize peripheral nociceptors and reduce proinflammatory cytokines peripherally and in the spinal cord [20, 21].

Studies on therapeutic application of TEAS in the management of spasticity are sparse, but the results from those studies consistently show the beneficial effects of TEAS on patients with spasticity following spinal cord injury or stroke [22–26]. Yan et al. conducted a randomized controlled trial to compare the effects of TEAS, placebo stimulation, and standard rehabilitation alone on muscle function after acute stroke and found that TEAS significantly increased the proportion of patients with improved muscle function [22]. Wang et al. evaluated the efficacy of 0, 2, and 100 Hz TEAS on spasticity after spinal cord injury and found 100 Hz TEAS relieved spasticity more efficiently and effectively than 2 Hz TEAS in patients [23]. Although the results are consistent, most of the studies except the report by Yan et al. are not randomized controlled trials, significantly compromising the quality of the data. Here, we conducted a randomized controlled trial to evaluate the clinical efficacy and safety of TEAS and compare the efficacy of high versus low TEAS frequency on patients with spasticity following brain injury.

## MATERIALS AND METHODS

The protocol for this trial, supporting CONSORT checklist, and the original data of the clinical trial are available as supporting information; see S1 Protocol, S1 CONSORT Checklist, and S1 Table.

### Trial design

**Figure 1:**
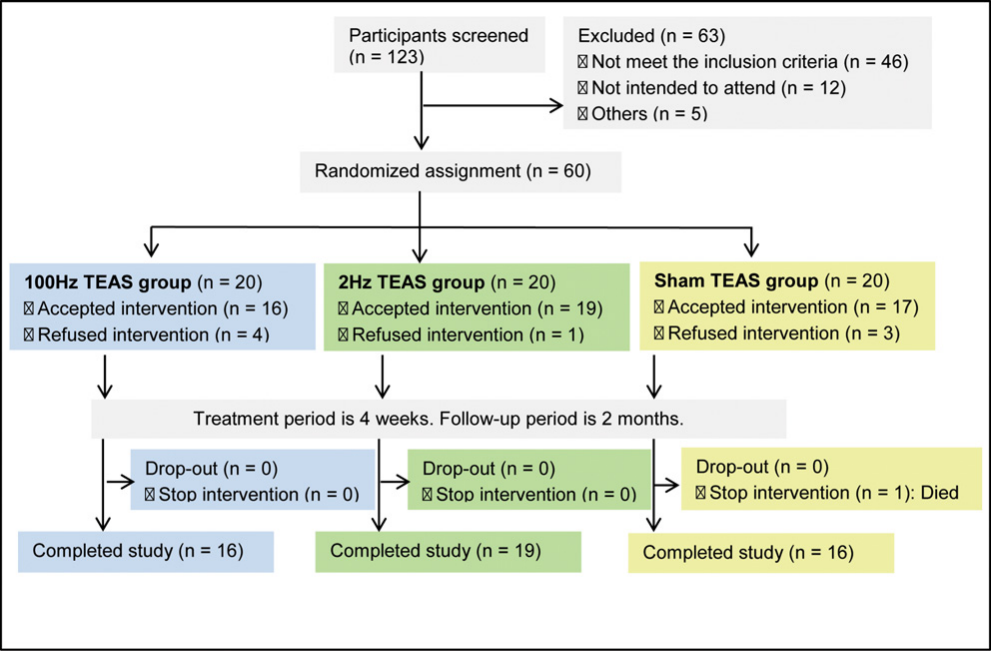
The CONSORT flow diagram.

**Figure 2:**
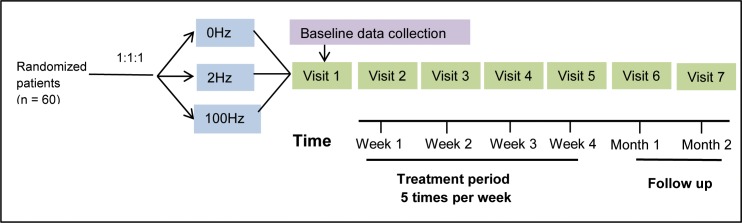
Study design.

This double-blinded, multicenter, randomized controlled trial was conducted at 6 clinical centers in the city of Tianjin and its surrounding counties between March and September 2011. This trial was conducted in accordance with the principles of the Declaration of Helsinki (Version Edinburgh 2000) and approved by the Chinese Ethics Committee of Registering Clinical Trial. The study was registered at the Chinese Clinical Trial Registry (http://www.chictr.org/cn, Unique Identifier: ChiCTR-TRC-11001310). The study protocol was approved by the Institutional Ethics Committee of Tianjin University of Traditional Chinese Medicine (TJUTCM-EC20110001). Written informed consent was obtained from all patients. The CONSORT flow diagram is displayed in Fig. 1. The trial design is illustrated in Fig. 2.

### Patients

Eligible patients were adults 18–85 years of age (both years inclusive) who did not receive previous TENS or TEAS treatments and had: experienced at least 1 episode of brain injury such as cerebral hemorrhage, cerebral infarction, or brain trauma (confirmed by examining patients’ medical records and computed tomography or magnetic resonance imaging of the brain) at least 3 months before study enrollment; increased muscle tone of the flexor carpi on the affected side with Modified Ashworth Scale (MAS) score ≥ 3; and disability score ≥ 2 according to the Disability Assessment Scale (DAS). Patients with the following conditions were excluded from the study: severe contracture at the joints of affected limbs (inability to passively move the joint by > 10°), previous Achilles tendon lengthening or transplantation surgery, previous nerve resection surgery, injection of botulinum toxin into the target limb within 4 months before enrollment, local neuromuscular blocking therapy or oral antispasmodic medication within 2 weeks before enrollment, severe muscle atrophy or infection in target limbs, pregnant or planning to become pregnant during the course of the study, or severe conditions in other organs such as severe liver and renal dysfunction or wearing a cardiac pacemaker.

### Randomization, allocation, and blinding

A central randomization scheme was used to assign eligible patients to 3 groups—100, 2, or 0 Hz TEAS (sham)—in a 1:1:1 ratio at the Tianjin University of Traditional Chinese Medicine. A permuted-block randomization procedure was used to generate an allocation sequence. The block size for randomization was 6. Sealed opaque envelopes containing the patient number and the allocated treatment were prepared. The envelopes were sequentially numbered. Several levels of blinding strategy were implemented to minimize possible bias. The personnel who recruited patients, professional assessors who evaluated patient performance, and the statistician were blinded to treatment allocation. The patients too, were completely blinded to the treatment administered. At the recruitment interview, the participants were informed that they were to undergo 1 of 3 new therapies for muscle spasticity. The following detailed explanations of the new therapies were also provided to the participants: “During treatment, acupuncture points will be stimulated by micro-currents generated by a special electrical device. The magnitude of the micro-currents is so low that a person might experience only mild electric current sensation or feel nothing at all.” To prevent any possible psychological interference on the results, the patients were allowed to watch the operation of the electrical stimulator and believed that they received real treatments. In the sham TEAS group, the electrodes were placed on the same acupoints and treated for the same duration as the other 2 groups. To further ensure the participants were blinded for treatments administered and to prevent any possible psychological interference, at each treatment session, a trained nurse who was blinded to treatment allocation was present and engaged a conversation with the participants to distract and help them relax.

As the acupuncturists had to administer treatment, it was impossible to completely blind them to patients’ information such as symptoms. However, the acupuncturists were forbidden to speak to patients during treatments. In the case that a patient expressed concerns or complains regarding treatments, the patient spoke to the nurse and the nurse forwarded the information to the acupuncturists, who would adjust the treatments. After setting up the TEAS instrument and ensuring the instrument worked properly, the acupuncturists left the treatment room. The unblinding was conducted in 2 steps. The first data unblinding was performed after the trial was completed and the database was locked. The statistician was exposed to the data organized according to treatment groups, represented as A, B, and C. The annotation of each letter was not revealed to the statistician. The second data unblinding was performed after data analyses were completed. All the information on patients and treatment allocation were then disclosed to investigators participating in the trial.

### Interventions

**Figure 3:**
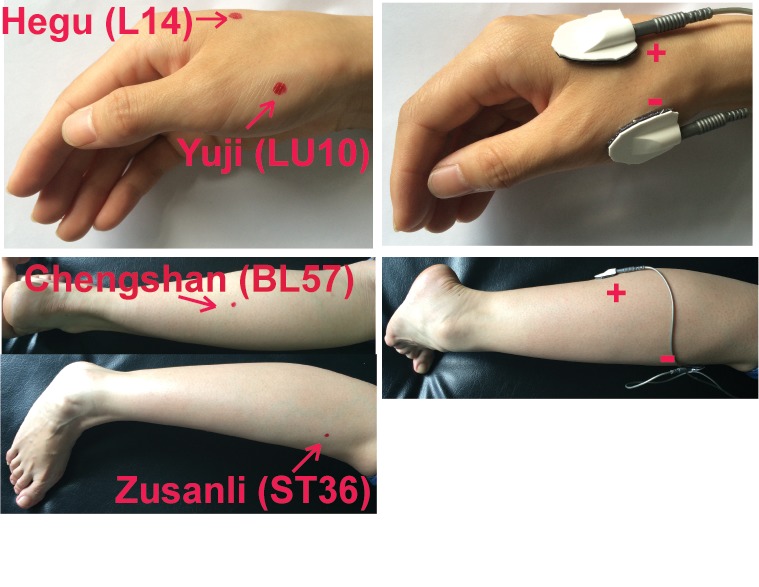
Location of the acupoints Hegu (L14)–Yuji (LU10) and Zusanli (ST36)–Chengshan (BL57).

All patients were allowed to continue their routine rehabilitation training and dietary regimen during the study. All eligible patients were also allowed to maintain their existing medication regimen as long as it did not violate the inclusion criteria. However, they were required to record details of their medication regimen throughout the study. A total of 2 electrical circuits were stimulated among acupoints using a TEAS instrument—the acupoint nerve electrical stimulator (HANS-100A, Nanjing Gensun medical technology company, Nanjing, China). The location of the acupoints is illustrated in Fig. 3. One pair of round percutaneous electrodes (diameter, 24 mm) was placed on the acupoints Hegu (LI4)–Yuji (LU10) on the affected side to induce an electric circuit. The other electric circuit was stimulated by placing another pair of electrodes on the acupoints Zusanli (ST36)–Chengshan (BL57) on the same side. These 2 pairs of electrodes were connected to the acupoint nerve stimulator. The acupoints were stimulated at 100 or 2 Hz for 0.2 ms. Frequencies were chosen based on the reports describing the therapeutic application and molecular mechanism of TEAS in spasticity [23, 27–29]. To treat patients receiving 100 or 2Hz TEAS, the acupuncturists gradually increased the intensity of the electrical stimulation to the level (usually between 20 and 40 mA) that rhythmic contraction of muscles on hands or legs was visible. Patients usually did not experience any pain or discomfort at such a level of intensity. The average intensity of the electrical stimulation used in this study was approximately 30 mA. Each treatment session lasted 30 minutes, and the patients received 5 treatment sessions (1 treatment session per day) every week for 4 weeks. In the sham TEAS group, the electrodes were placed on the same acupoints and treated for the same duration as the other 2 groups; however, the intensity of electric stimulation was 0 mA. All patients were allowed to watch the operation of the electrical stimulator and to view the entire procedure during treatment sessions, and believed they received real treatments.

### Assessments

Patients were required to complete the questionnaires weekly after the fifth treatment every week and at the follow-up visits 1 and 2 months after the treatment. The severity of muscle spasticity and disability, walking capability, and overall performance of daily activity of patients were assessed by a professionally trained assessor at enrollment, after the fifth treatment every week, and at each follow-up visit. MAS was used to assess muscle tone (0 represents no increase in muscle tone; 4 represents the highest increase in muscle tone with rigidity in flexion or extension) [30]. Disability was assessed by DAS on a scale of 0–3, with 0 indicating normal and 3 indicating severe disability [31]. Walking capability was evaluated by the Holden functional ambulation classification score on a scale of 1–5, with higher scores indicating better walking capability [32], whereas overall performance was assessed by the Global Assessment Scale (GAS) score at a range of 1–100 with higher scores suggesting a better overall level of functioning and carrying our activities of daily living [33] and the improved Barthel Index (BI) score in the range of 1–100 with higher scores indicating a better performance in activities of daily living [34, 35]. We also compared the percentage of patients versus the percentage of caregivers expecting post-treatment improvement in daily activities that were adversely influenced by disability.

### Endpoints

The primary endpoints included the MAS score of the wrist, thumb, and the other 4 fingers. The secondary endpoints were the MAS score of the elbow, shoulder, knee, and ankle, the DAS score of disability, Holden functional ambulation classification score, GAS score, and improved BI scores. Adverse event was defined as an occurrence of any of the following events: bleeding, hematoma, syncope, severe pain, and local infection. Adverse events were monitored during the study and documented during treatment and at follow-up visits.

### Sample size calculation and statistical analysis

Based on our previous study, to detect a significant difference at the level of 0.05 between the groups with 90% power at the assumption of a drop-out rate of 15% and a 1-tailed α = 0.05, 20 patients (total 60 patients) were required for each treatment group [23]. Data were presented as mean ± standard deviation (SD). Count variables with normal distribution were analyzed by one-way or repeated-measures ANOVA to examine differences and by Pearson’s chi-square test to determine correlation. Count variables with partial distribution were analyzed using a rank-sum or Spearman rank correlation test. For baseline general clinical characteristics, one-way ANOVA was performed on continuous variables including age, spasticity duration, height, weight, and blood pressure, whereas Pearson’s chi-square test was used on count variables including gender, brain injury type, and comorbid disease. One-way ANOVA was also performed on the baseline primary (MAS score of the wrist, thumb, and the other 4 fingers) and secondary endpoints (DAS score, Holden functional ambulation classification score, GAS score, and improved BI scores). The correlations among baseline endpoints were analyzed by Pearson’s chi-square test. To assess treatment efficacy, data of the primary and secondary endpoints were examined by Mauchly’s sphericity test first, and then repeated-measures ANOVA was performed to analyze the data. All statistical analyses were conducted on the intention-to-treat (ITT) population. The primary endpoints from per-protocol (PP) datasets were also analyzed using the same statistical analyses. The ITT set included all the enrolled patients (*n* = 60); the PP set included patients in the ITT set who did not deviate from the protocol. In total, 9 patients (15%) dropped out of the study, including 8 not receiving treatment and 1 died from an infectious complication unrelated to treatments. Thus, the PP set included 51 patients. *P* < 0.05 (2-sided) was considered statistically significant. All analyses were performed using the SPSS 18.0 software.

## RESULTS

### Baseline clinical data are similar among the treatment groups

Of the 123 patients screened, 60 were randomized, and 51 completed the study. The patient flowchart is shown in Fig. 1. The majority of patients were male; the mean age of the patients included in the study was > 60 years in all 3 groups. The baseline characteristics of the patients were comparable across all 3 treatment groups (*P* > 0.05; Table 1). Additionally, the baseline muscle spasticity at the wrist, thumb, the other 4 fingers, elbow, shoulder, knee, and ankle evaluated by the MAS score, the severity of disability reflected by DAS score, walking capability evaluated by Holder functional ambulation classification, and overall performance reflected by the GAS score and improved BI were not significantly different among the 3 groups (*P* > 0.05, Table 2).

**Table 1:** Baseline characteristics.

Characteristic	100 Hz group	2 Hz group	Sham group
Age (years)	62.00 ± 9.20	63.50 ± 9.29	62.45 ± 8.44
Gender, n			
Male	15	16	15
Female	5	4	5
Brain injury type, n			
Cerebral infarction	15	15	17
Trauma	0	1	0
Hemorrhage	5	4	3
Comorbid disease, n			
Yes	14	9	11
No	6	11	9
Spasticity duration (years)	5.758 ± 5.174	4.717 ± 5.091	3.495 ± 3.527
Height (cm)	171.70 ± 7.47	170.85 ± 6.35	169.75 ± 7.33
Weight (kg)	69.33 ± 8.35	66.40 ± 8.46	66.15 ± 8.94
Systolic pressure (mmHg)	136.35 ± 12.487	138.50 ± 8.599	135.25 ± 12.298
Diastolic pressure (mmHg)	90.20 ± 8.383	88.50 ± 6.091	86.25 ± 9.301

Data are presented as mean ± standard deviation unless otherwise indicated. One-way ANOVA was performed on continuous variables including age, spasticity duration, height, weight, and blood pressure; the Chi-square test was used on count variables including gender, brain injury type, and comorbid disease. All variables were not significantly different among the 3 groups (*P* > 0.05).

**Table 2:** Baseline MAS score of the wrist, thumb, the other 4 fingers, elbow, shoulder, knee, and ankle, DAS score, walking capability, and overall performance.

	100 Hz group	2 Hz group	Sham group
MAS score			
Wrist	3.150 ± 0.366	3.200 ± 0.410	3.100 ± 0.308
Thumb	2.400 ± 0.868	2.550 ± 0.742	2.450 ± 0.686
Other 4 fingers	2.625 ± 0.944	2.525 ± 0.638	2.525 ± 0.896
Elbow	2.900 ± 0.837	2.575 ± 0.950	2.450 ± 0.985
Shoulder	1.725 ± 1.019	1.525 ± 0.819	1.725 ± 0.678
Knee	2.200 ± 0.938	2.175 ± 0.766	1.800 ± 0.571
Ankle	2.675 ± 0.694	2.775 ± 0.734	2.900 ± 0.718
DAS score			
Personal hygiene	2.800 ± 0.410	2.850 ± 0.366	2.750 ± 0.550
Dressing	2.750 ± 0.444	2.850 ± 0.366	2.750 ± 0.444
Posture	2.750 ± 0.550	2.750 ± 0.444	2.650 ± 0.587
Pain or discomfort	1.350 ± 1.040	1.300 ± 0.657	1.450 ± 0.759
Holder functional ambulation classification	3.050 ± 1.432	2.900 ± 1.917	2.800 ± 1.542
GAS score	56.65 ± 15.83	55.00 ± 16.16	53.40 ± 11.27
Improved Barthel Index	65.15 ± 23.94	64.85 ± 26.78	68.60 ± 22.32

Data are presented as mean ± standard deviation. MAS = Modified Ashworth Scale. DAS = Disability Assessment Scale. GAS = Global Assessment Scale. One-way ANOVA was performed on the data. All the variables were not significantly different among the 3 groups (*P* > 0.05).

We then analyzed the possible association of muscle spasticity at the wrist and fingers with the capability of daily activities and the correlation of muscle spasticity at the knee and ankle with walking capability in our patients. The results show significant correlation of muscle spasticity at the wrist, thumb, and the other 4 fingers with each other and with the capabilities for personal hygiene and posture (*P* < 0.01, Table 3). In contrast, the MAS score of the knee and ankle did not seem to be associated with the walking capability. As both GAS and improved BI indicate overall performance, it is not surprising that these 2 values were closely associated with each other. Because improvement in activity for personal hygiene was the key expectation of both patients and caregivers (S1 Fig.) and wrist and hand function strongly correlated with this activity, we focused on the improvement of wrist and hand spasticity in this study.

**Table 3:** Correlation of wrist and hand function with activity capability before treatment.

Analyzed parameters		Pearson coefficient	*P* value (2 sides)
Wrist (MAS score)	Thumb (MAS score)	0.360	**0.005** **
Wrist (MAS score)	Other 4 fingers (MAS score)	0.227	0.081
Thumb (MAS score)	Other 4 fingers (MAS score)	0.403	0.001**
Thumb (MAS score)	Personal hygiene (DAS score)	0.333	0.009**
Other 4 fingers (MAS score)	Personal hygiene (DAS score)	0.357	0.005**
Other 4 fingers (MAS score)	Posture (DAS score)	0.432	0.001**
Thumb (MAS score)	Dressing (DAS score)	0.165	0.208
Other 4 fingers (MAS score)	Dressing (DAS score)	0.211	0.106
Other 4 fingers (MAS score)	GAS	-0.240	0.065
Wrist (MAS score)	Improved Barthel Index	-0.226	0.083
Other 4 fingers (MAS score)	Improved Barthel Index	-0.312	0.015*
GAS	Improved Barthel Index	0.518	< 0.0001**
Knee (MAS score)	Holden functional ambulation classification	-0.170	0.193
Ankle (MAS score)	Holden functional ambulation classification	-0.119	0.364
Knee (MAS score)	Improved Barthel Index	-0.201	0.124
Ankle (MAS score)	Improved Barthel Index	-0.055	0.676

Data were analyzed by Pearson’s chi-square test.

*P 0.05.

**P 0.01.

MAS = Modified Ashworth Scale. DAS = Disability Assessment Scale. GAS = Global Assessment Scale.

### Efficacy

**Figure 4:**
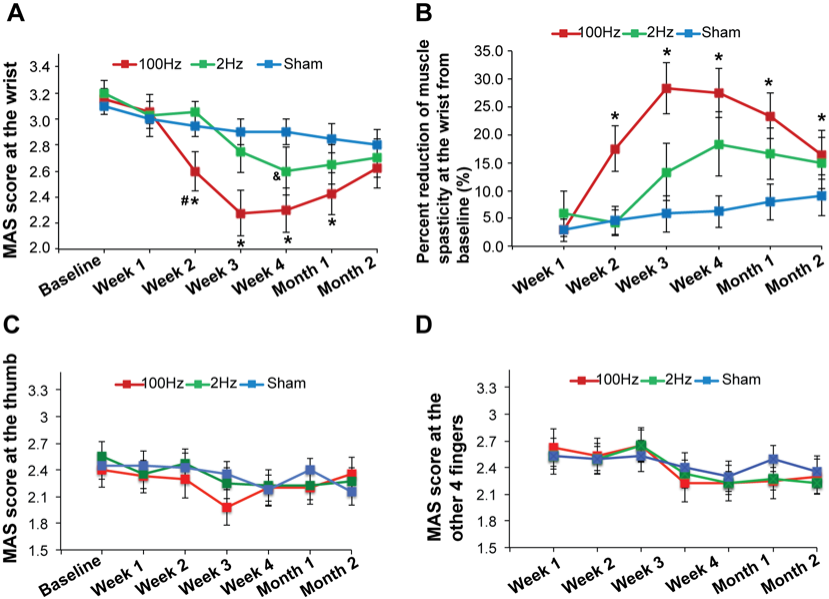
Efficacy of 100 Hz, 2 Hz, or sham TEAS. **A.** Wrist MAS score was significantly reduced by 100 Hz TEAS compared with 2 Hz or sham TEAS. Data were analyzed by repeated-measures ANOVA. The * represents significant difference between 100 Hz and sham, *P* < 0.05; ^#^ represents significant difference between 100 Hz and 2 Hz, *P* < 0.05; ^&^ represents significant difference between 2 Hz and sham. **B**. Wrist MAS score in patients treated with 100 Hz TEAS was significantly decreased from baseline during treatment and at follow-up visits. Comparison with baseline values was analyzed by repeated-measures ANOVA. The wrist MAS scores were used for the statistical analysis. Data were presented as mean ± standard error. The * represents significant differences between post-treatment and the baseline value, *P* < 0.05. **C**. Thumb MAS score was not affected by treatments. **D.** The MAS score of the other 4 fingers was not affected by treatments. Results are from the analysis on ITT data set. ANOVA = analysis of variance. MAS = Modified Ashworth Scale. TEAS = transcutaneous electrical acupoint stimulation.

**TEAS with 100 Hz stimulation significantly reduces wrist spasticity.** Compared with sham treatment, 100 Hz TEAS significantly reduced wrist MAS score at weeks 2, 3, and 4 of the treatment, and 1 month after the treatment, whereas 2 Hz stimulation only decreased wrist MAS score significantly at Week 4 of the treatment (*P* < 0.05; Fig. 4A). In addition, TEAS 100 Hz significantly reduced wrist MAS score from baseline at weeks 2, 3, and 4 of treatment and at the 1- and 2-month follow-up visits (*P* < 0.05; Fig. 4B). The maximal reduction in MAS score at the wrist from baseline (28.3% ± 4.54%) was observed at Week 3 of the 100 Hz treatment (Fig. 4B). These results suggest that wrist spasticity following brain injury can be substantially attenuated by 100 Hz TEAS. Neither 100 Hz nor 2 Hz treatment affected the MAS score of the thumb (Fig. 4C) and the other 4 fingers (Fig. 4D). The results from repeated-measures ANOVA also show that, in addition to the intervention (*P* = 0.012) and time (*P* < 0.001), the interaction between time and intervention also significantly affected wrist MAS score (*P* = 0.003), indicating that the rate of change in the MAS score over time is different for each treatment group. The MAS score at the other 4 fingers was only significantly affected by time (*P* < 0.001). Time, intervention, or interaction between time and intervention did not affect thumb MAS score significantly.

### Secondary endpoints

None of the secondary endpoints, including the MAS score for the elbow, shoulder, knee, and ankle, DAS score, Holden functional ambulation classification score, GAS score, and the improved BI score were changed with 100 Hz or 2 Hz treatments (Tables 4,5,6). Time or the interaction of time and intervention did not affect the secondary endpoints significantly.

**Table 4:** MAS score of the elbow, shoulder, knee, and ankle.

		MAS Elbow	MAS Shoulder	MAS Knee	MAS Ankle
Baseline	100 Hz	2.3±1.01	1.5±1.03	1.88±0.67	2.68±0.95
	2 Hz	2.6±0.84	1.8±0.62	2.0±0.72	2.8±0.52
	Sham	3±0.84	1.725±0.85	2.35±0.81	2.875±0.6
Week 1	100 Hz	1.83±0.0.77	1.53±1.04	1.83±0.88	2.7±0.91
	2 Hz	2.53±0.97	1.75±0.53	2±0.69	2.8±0.68
	Sham	2.75±0.7	1.825±0.73	2.35±0.86	2.9±0.55
Week 2	100 Hz	1.75±0.72	1.25±0.88	1.575±0.8	2.23±0.94
	2 Hz	2.55±0.87	1.68±0.65	1.88±0.70	2.73±0.6
	Sham	2.6±0.74	1.78±0.83	2.15±0.86	2.88±0.6
Week 3	100 Hz	1.9±0.64	1.38±0.9	1.78±0.82	2.38±0.96
	2 Hz	2.35±0.84	1.55±0.63	1.85±0.71	2.65±0.65
	Sham	2.33±0.8	1.6±0.85	2.08±0.78	2.85±0.59
Week 4	100 Hz	2±0.73	1.33±0.91	1.65±0.83	2.38±0.81
	2 Hz	2.25±0.7	1.7±0.7	1.73±0.5	2.53±0.7
	Sham	2.4±0.79	1.7±0.85	2.05±0.65	2.7±0.57
Month 1	100 Hz	2±0.76	1.25±0.87	1.75±0.94	2.45±0.79
	2 Hz	2.35±0.78	1.7±0.5	1.8±0.66	2.7±0.7
	Sham	2.48±0.77	1.68±0.73	2.13±0.69	2.78±0.57
Month 2	100 Hz	2.2±0.68	1.43±0.98	1.78±0.88	2.55±0.74
	2 Hz	2.35±0.83	1.53±0.55	1.73±0.47	2.7±0.55
	Sham	2.6±0.66	1.83±0.75	2±0.71	2.78±0.57

Data were analyzed by repeated-measures ANOVA. MAS = Modified Ashworth Scale.

**Table 5:** DAS score of personal hygiene, dressing, posture, and pain.

		DAS Hygiene	DAS Dressing	DAS Posture	DAS Pain
Baseline	100 Hz	2.55±0.6	2.6±0.5	2.45±0.69	1±0.73
	2 Hz	2.9±0.37	2.9±0.37	2.8±0.41	1.5±0.69
	Sham	3±0.001	2.9±0.31	2.9±0.31	1.65±0.93
Week 1	100 Hz	2.55±0.69	2.55±0.60	2.45±0.69	1±0.56
	2 Hz	2.7±0.57	2.7±0.57	2.7±0.57	1.3±0.73
	Sham	3±0.001	2.9±0.31	2.9±0.31	1.7±0.8
Week 2	100 Hz	2.75±0.44	2.65±0.59	2.5±0.76	0.7±0.57
	2 Hz	2.8±0.41	2.85±0.37	2.65±0.49	1.2±0.7
	Sham	2.95±0.22	2.9±0.31	2.85±0.37	1.65±0.75
Week 3	100 Hz	2.65±0.59	2.65±0.59	2.4±0.82	0.95±0.39
	2 Hz	2.75±0.44	2.7±0.47	2.6±0.6	1.25±0.55
	Sham	2.85±0.37	2.8±0.41	2.75±0.44	1.45±0.94
Week 4	100 Hz	2.65±0.49	2.6±0.50	2.35±0.67	0.65±0.81
	2 Hz	2.65±0.59	2.6±0.6	2.4±0.82	1.35±0.67
	Sham	2.9±0.31	2.85±0.37	2.8±0.37	1.55±0.83
Month 1	100 Hz	2.55±0.76	2.4±0.75	2.15±0.67	0.95±0.6
	2 Hz	2.8±0.41	2.7±0.57	2.6±0.68	1.3±0.66
	Sham	2.9±0.31	2.85±0.37	2.75±0.55	1.35±0.81
Month 2	100 Hz	2.65±0.49	2.6±0.5	2.35±0.67	0.75±0.55
	2 Hz	2.8±0.41	2.65±0.67	2.55±0.76	1.25±0.72
	Sham	2.95±0.22	2.85±0.37	2.8±.41	1.4±0.82

Data were analyzed by repeated-measures ANOVA. DAS = Disability Assessment Scale.

**Table 6:** Holden functional ambulation classification score, GAS score, and the improved Barthel Index score.

		Holden Score	GAS Score	Improved Barthel Index
Baseline	100 Hz	2.7±1.49	52.05±9.76	66.05±25.34
	2 Hz	3.3±1.37	57.4±11.54	72.6±15.59
	Sham	2.8±1.96	55.6±20.01	60±28.79
Week 1	100 Hz	2.8±1.51	53.15±10.01	67.35±25.99
	2 Hz	3.15±1.42	60.1±13.18	70.7±16.26
	Sham	2.8±1.96	58±18.94	60.25±29.15
Week 2	100 Hz	2.8±1.51	53.75±5.44	79.45±17.04
	2 Hz	3.15±1.42	60.35±10.33	69.75±20.40
	Sham	2.8±1.96	59.1±18.21	60.4±28.35
Week 3	100 Hz	3.05±1.57	54.6±6.18	72.15±24.51
	2 Hz	3.05±1.47	60.5±10.58	69.55±18.62
	Sham	2.85±1.93	60.2±17.98	61.1±28.30
Week 4	100 Hz	3.1±1.59	55.3±6.95	72.8±23.17
	2 Hz	3.1±1.48	59.75±11.92	68.2±20
	Sham	2.85±1.93	61.2±18.33	61±28.64
Month 1	100 Hz	3.1±1.41	56.9±7.1	72.65±23.57
	2 Hz	3.1±1.55	61.2±12.57	71.55±20.12
	Sham	2.8±1.94	62.3±18.49	63.4±28.02
Month 2	100 Hz	3.15±1.42	59.3±8.27	73.6±21.49
	2 Hz	3.1±1.55	61.2±12.77	73.35±19.07
	Sham	2.75±1.92	63.85±16.58	63.65±28.19

Data were analyzed by repeated-measures ANOVA. GAS = Global Assessment Scale.

### Safety

No treatment-emergent adverse events such as bleeding, hematoma, syncope, severe pain, or local infection were reported by the patients during treatment and follow-up visits. One patient died from complications associated with cerebral stroke, which were not related to the treatments in this trial.

## DISCUSSION

In this study, we found that 100 Hz TEAS treatment significantly reduced wrist spasticity from baseline in a multicenter, double-blinded, randomized controlled trial. Our results also show that, compared with 2 Hz TEAS, 100 Hz stimulation more effectively attenuated wrist spasticity at weeks 2, 3, and 4 of treatment and at the 1-month follow-up visit. Although our analysis on the ITT dataset reveals that 2 Hz stimulation also significantly reduced muscle wrist spasticity at Week 4 of the treatment compared with sham treatment, the analysis on the PP dataset did not find any statistically significant difference between the 2 Hz and sham groups at each visit (data not shown).

Our findings are consistent with results from the previous study demonstrating that 100 Hz TEAS can more effectively relieve muscle spasticity in patients with spinal cord injury than 2 Hz TEAS [23, 25–27]. TEAS at 100 Hz also relieved muscle spasticity in a rat model [36]. In contrast, studies on patients or animal models demonstrate that 2 Hz TEAS or EA seems more effective than 100 Hz when used for other medical conditions such as inflammation, pain, and motor dysfunction after ischemic stroke [20, 21]. The disease-dependent difference in the effects of high versus low frequency of TEAS suggests that different mechanisms are involved in spasticity versus pain. Studies conducted in human and animal models demonstrate that 2 Hz TEAS or EA stimulates the levels of enkephalins and endorphins in cerebrospinal fluid, whereas 100 Hz induces the release of dynorphins [20, 27, 28]. Thus, our observation that 100 Hz but not 2Hz TEAS exerted beneficial effects in patients with spasticity indicates that dynorphins might play a role in spasticity. Indeed, the study on a rat model of spasticity induced by spinal cord injury shows that the levels of dynorphin A (1–17) decrease significantly in the thoracic and lumbar segments of the spinal cord in the rat and that stimulation of the kappa opioid receptor by the agonist U-50, 488H relieves muscle spasticity [36], further supporting a possible role of dynorphins in mediating spasticity. Furthermore, Wang et al. found that inhibition of opioid receptors by the antagonist naloxone could partially block the 100 Hz electrical stimulation-mediated spasmolytic effects in patients [23]. Thus, 100 Hz TEAS might relieve spasticity after brain injury by stimulating the release of dynorphins or inducing the activity of opioid receptors.

In our study, 100 Hz TEAS reduced muscle spasticity only at the wrist, but did not affect muscle spasticity at the thumb and in the other 4 fingers although the MAS score of the 3 areas correlated with each other significantly. This may be attributable to the relatively small sample size in this study. Our sample size estimation, which was based on the previous study that focused on muscle spasticity at the wrist [23], may have been inadequate. Additionally, finger movement requires fine motor skill; therefore, a treatment period longer than 4 weeks might be required to produce significant improvements. The beneficial effects of 100 Hz TEAS on reduction of wrist spasticity appear to be treatment dependent. At Week 3 of 100 Hz TEAS, the reduction of wrist spasticity from baseline reached its peak and declined substantially 2 months after treatment. Nevertheless, by the end of the study, wrist spasticity in the 100 Hz group showed a significant decrease of approximately 15% from baseline. These results further suggest that a longer period of treatment might produce better efficacy. The acupoints in this study were selected based on the previous report on TEAS treatment for muscle spasticity after spinal cord injury [23]. As the optimal acupoints for treatment of spinal cord injury-associated spasticity might not be ideal for treating spasticity following brain injury, additional acupoints should be included in future studies. Study limitations such as small sample size, short treatment period, and suboptimal acupoint selection might also contribute to the absence of significant beneficial effects of 100 Hz treatment on the secondary endpoints including MAS score at the elbow, shoulder, knee, and ankle, severity of disability (DAS score), walking capability (Holden functional ambulation classification score), and overall performance (GAS and improved BI scores).

TEAS seems to be a safer and more standardized approach than conventional acupuncture because of the avoidance of acupuncture needles and reduced dependence on the acupuncturist’s experience. Compared with other therapies for muscle spasticity, TEAS appears to be more convenient for patients because it allows self-treatment in the comfort of one’s home, and the instrument can be easily operated. Our results indicate that TEAS might be a promising therapy to reduce muscle spasticity following brain injury and eventually improve the quality of life of patients. Future research is aimed at conducting a larger clinical trial with longer treatment duration to further investigate the effects of TEAS on muscle spasticity after brain injury.

## CONCLUSION

Our results suggest that TEAS might be a safe and effective therapy to relieve muscle spasticity following brain injury; however, the findings need to be further verified by larger scale studies.

## SUPPORTING INFORMATION

S1 Fig Percentage comparison of patients’ versus caregivers’ expectation of improvements in activities adversely affected by disability. The majority of patients and caregivers (> 50%) expected an improvement in daily activities related to personal hygiene after treatment. This indicates that personal hygiene is considered as the most important daily activity by both patients and caregivers. Although a similar proportion of patients and caregivers expected improvement in dressing activity and posture, their views on pain were quite different. Interestingly, a significantly higher proportion of patients hoped that treatment would reduce pain compared with caregivers (18% versus 3%).
(TIF)

S1 CONSORT Checklist The filled CONSORT checklist for this study. (DOC)

S1 Protocol The study protocol for this clinical trial. (DOC)

S1 Table Original trial data. (XLSX)
