# Supplementary figures and images for: Efficacy and Safety of Transcutaneous Electrical Acupoint Stimulation to Treat Muscle Spasticity following Brain Injury: A Double-Blinded, Multicenter, Randomized Controlled Trial
Source: PLoS One. 2015 Feb 2;10(2):e0116976. doi: 10.1371/journal.pone.0116976 (PMC4314074; doi:10.1371/journal.pone.0116976)

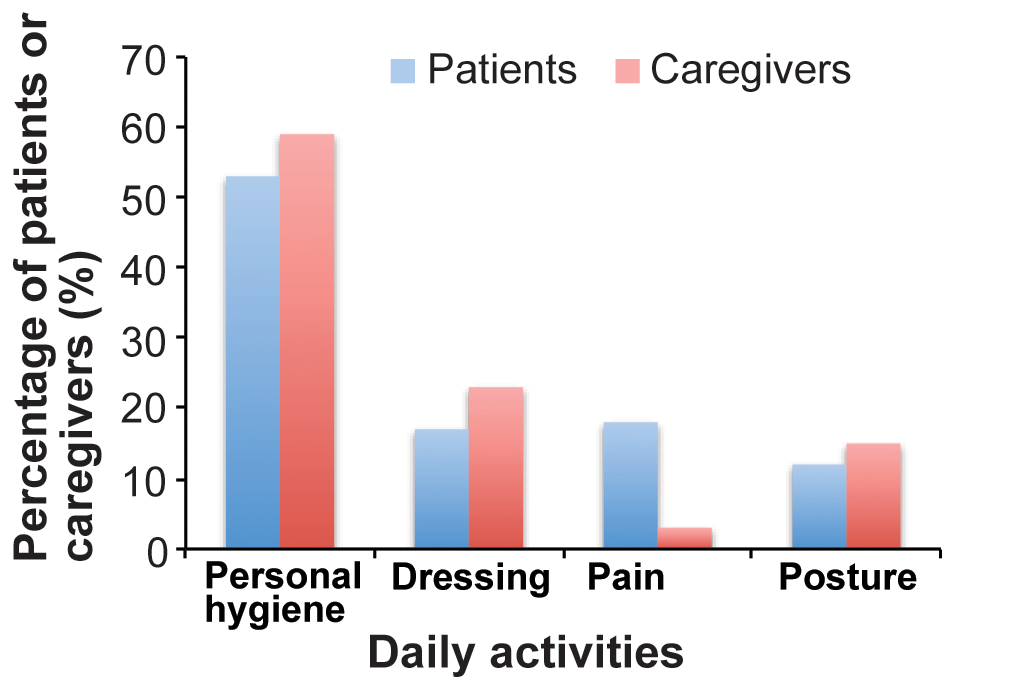

Supplement: S1 Fig — The majority of patients and caregivers (> 50%) expected an improvement in daily activities related to personal hygiene after treatment. This indicates that personal hygiene is considered as the most important daily activity by both patients and caregivers. Although a similar proportion of patients and caregivers expected improvement in dressing activity and posture, their views on pain were quite different. Interestingly, a significantly higher proportion of patients hoped that treatment would reduce pain compared with caregivers (18% versus 3%). (TIF) [file pone.0116976.s001.tif]
