# Supplementary material for: Efficacy and Safety of Transcutaneous Electrical Acupoint Stimulation to Treat Muscle Spasticity following Brain Injury: A Double-Blinded, Multicenter, Randomized Controlled Trial
Source: PLoS One. 2015 Feb 2;10(2):e0116976. doi: 10.1371/journal.pone.0116976 (PMC4314074; doi:10.1371/journal.pone.0116976)
Supplement: S1 Protocol — (DOC) [file pone.0116976.s003.doc]

**A randomized controlled clinical study: Transcutaneous acupoint electrical stimulation in the treatment of muscle spasm**

Applicant: Ye ZHAO

Unit: Acupuncture College, Tianjin University of Traditional Chinese Medicine

Contact address: 312 Anshan West Avenue, Nankai District, Tianjin

Tel: 13662121309 E-mail: [zhaoye@tjutcm.edu.cn](mailto:zhaoye@tjutcm.edu.cn)

Fax: 022-27485189 PC: 300193

Study period: December 2010 - May 2012

Written in 2010

**Professional experience of the lead authors**

| Mr. Ye Zhao is pursuing PhD in acupuncture and massage at the Tianjin University of Traditional Chinese Medicine, with the primary focus on acupuncture-related clinical experimental study. From September 2002 to July 2005, he majored in basic integrated Chinese and Western medicine at the Tianjin University of Traditional Chinese Medicine, and later graduated with a master’s degree in medicine. The title of his dissertation is “impact of pricking blood therapy of well points on the degeneration of neural stem cells after focal cerebral ischemia injuries in rats.” From July 2005 to July 2008, he worked at the Department of Integrated Chinese and Western Medicine at Tianjin Children’s Hospital as a physician.  Jisheng Han is a professor, a doctoral supervisor, and an academician at the Chinese Academy of Sciences, and the honorary director of the Neuroscience Research Centre at Peking University. He has conducted long-term research in acupuncture analgesia, being responsible for clarifying its neurochemical mechanisms, and won a third prize for National Natural Science in 1987. With this as the basis, he further clarified the physiological and molecular mechanisms of how different frequencies of electro-acupuncture could cause the release of a variety of neuropeptides, for which he received the second prize for National Natural Science in 1999. In cooperation with Professor Yiming Liu from the Beijing University of Aeronautics and Astronautics, Professor Han successfully developed Han’s acupoint nerve stimulator (HANS) that can be used in the treatment of digitized electro-acupuncture and transcutaneous neural stimulation. On August 2010, he was asked to present a report on acupuncture analgesia at the 13^th^ World Congress on Pain.  Yi Guo is a professor, a doctoral supervisor, and the dean of the Acupuncture College of Tianjin University of Traditional Chinese Medicine. He was in charge of the research subject in the national “eighth five-year plan” in ascending meridians, the national “eighth five-year plan” breakthrough, the national “tenth five-year plan” breakthrough, the national “973” sub-project, the National Natural Science Key Foundation, the National Natural Science Foundation, the Henry Fok Young Teacher Foundation in the Ministry of Education, as well as more than 50 ministerial and municipal projects. He took charge and participated in the formulation of 5 national acupuncture protocols which have been released. |
| --- |

Content

[Abstract 1](#__RefHeading___Toc395177471)

[**1．** **Background and significance of study** 1](#__RefHeading___Toc395177472)

[**2．** **Current domestic and international studies and foundation of research group’s early-stage work** 2](#__RefHeading___Toc395177473)

[**3．** **Detailed clinical study** 3](#__RefHeading___Toc395177474)

[3.1 Study objectives 3](#__RefHeading___Toc395177475)

[3.2 Design of study 4](#__RefHeading___Toc395177476)

[3.2.1 Source of study subjects 4](#__RefHeading___Toc395177477)

[3.2.2 Grouping and randomization of study subjects 4](#__RefHeading___Toc395177478)

[3.2.3 Blinding method and baseline date collection prior study 5](#__RefHeading___Toc395177479)

[3.2.4 Estimation of sample size and number of clinical study centers 5](#__RefHeading___Toc395177480)

[3.2.5 Selection of study subjects 8](#__RefHeading___Toc395177481)

[3.2.6 Treatment regimen 10](#__RefHeading___Toc395177482)

[3.2.7 Assessment system 11](#__RefHeading___Toc395177483)

[3.2.8 Quality control 16](#__RefHeading___Toc395177484)

[3.2.9 Management of adverse events 17](#__RefHeading___Toc395177485)

[3.2.10 Ethical clearance from the committee and registration of clinical study 17](#__RefHeading___Toc395177486)

[3.2.11 Statistical analysis 18](#__RefHeading___Toc395177487)

[**4．** **Study design** 20](#__RefHeading___Toc395177488)

[**5．** **References** 21](#__RefHeading___Toc395177489)

# Abstract

Spasm is one of the clinical signs associated with cerebral and spinal cord upper motor neuron lesions. According to reports, approximately 20%-40% of stroke patients 3 months after the onset of disease, 60% of patients with severe multiple sclerosis (MS), and 75% of the physically disabled patients with severe traumatic brain injuries will suffer from muscle spasms that require immediate treatment. Early intervention of severe muscle spasm can help avoid impairment from secondary functional damage and loss of mobility. Since the current surgical approach only relieves spasm while causing damage to normal function, it is not, therefore, generally used as the preferred treatment of choice. Drug treatment may relieve spasm; however, the drugs are usually short-acting and reduce muscle power. The use of drug therapy can also be further limited by the potential adverse effects of sedation, respiratory suppression, hallucinations, and prolonged QT intervals on ECG when used in large doses. Based on previous work, we propose to undertake a multicenter, large sample size, randomized, double-blind, parallel-group, placebo-controlled clinical study to assess the clinical efficacy and safety of transcutaneous acupoint electrical stimulation in treating patients with limb spasm after a cerebral vascular event and brain trauma, to provide objective evidence to support the clinical promotion of this therapy.

1. **Background and significance of study**

Spasticity is “increased, involuntary, velocity-dependent muscle tone that causes resistance to movement in patients with upper motor neuron lesions.”^1^ The upper motor neurons are located in the brain and spinal cord; damage to these neurons can result from a stroke, MS, spinal cord injury, brain injury, cerebral palsy, and other brain or spinal cord lesions. Its clinical presentation is increased muscle tone, brisk or even hyper deep tendon reflexes, caused by a lack of central inhibition. It has been estimated that approximately 20%-40% of stroke patients 3 months^2^ after the onset of disease, 60% of patients with severe MS, and as many as 75% of physically disabled patients with severe traumatic brain injuries will experience muscle spasms that require intervention.^3^

The presentation of spasms varies in individual patients; indeed, minor spasms can be beneficial. In spasticity, muscles do not become atrophic, which can effectively prevent deep venous thrombosis, limb edema, and osteoporosis. For paraplegic patients, some degree of spasm can help to maintain posture; move, stand, or even walk; as well as prevent pressure sores. When spasms affect daily activities, gait, sleep, or the personal hygiene of patients, or when spasms cause severe pain and contractions, they require progressive intervention. If severe muscle spasms are not managed properly, a vicious cycle will result, in which the muscles involved become ineffective against contractions caused by spastic dystonia, and this will lead to abnormal limb posture, which can further result in the shortening of soft tissues and biomechanical changes in the contracting muscles. These changes can, in turn, prevent muscle elongation, which can then worsen the dystonia. Early intervention in muscle spasms can prevent secondary maladjustment and functional damage, and avoid loss of mobility or of the ability to take part in activities.^4^

1. **Current domestic and international studies and foundation of research group’s early-stage work**

Nowadays, there are several main treatment approaches for spasms in this country as well as overseas. Invasive and irreversible surgical techniques, such as transecting nerves, lengthening of tendons, and transplantation of tendons, relieve spasm whilst at the same time compromise normal function; therefore, this approach is not usually the preferred method of treatment.^5^ Drugs such as baclofen, tizanidine, and benzodiazepine have some ability to relieve spasms, but these drugs are often short-acting and may reduce muscle power. Less satisfactory improvement in the overall neural function of the patients and the potential severe side effects of sedation, respiratory difficulties, hallucinations, and prolonged QT interval on ECG when used in large doses further limits their wide application.^6^ Botulinum toxin injection is a more popular treatment. Nevertheless, its main use in relieving local and small area spasms limits its wider clinical use.^7^ Compared with the methods above, trans-cutaneous electrical nerve stimulation, which involves placing electrodes on the skin surface to stimulate the nerves, is characterized as a simple and harmless procedure with no side effects.^8^ In our early-stage clinical study, it has been proved that when treating spasms caused by spinal cord lesions with transcutaneous acupoint electrical stimulation, the effects of high frequency (100 Hz) stimulation are apparently faster-acting and longer-lasting, when compared with lumbar and sacral stimulation; the benefits of treatment by acupoint stimulation last longer.^9^

Based on the work of early-stage transcutaneous acupoint electrical stimulation in the treatment of spasms due to spinal cord lesions, our research proposal is for a multicenter, large sample size, randomized, placebo-controlled, double-blind clinical study, to observe patients who develop limb spasms during the period following stroke and brain trauma. Using the modified Ashworth score, eligible patients will be randomized into a test group of transcutaneous acupoint high frequency (100 Hz) electrical stimulation, a test group of transcutaneous acupoint low frequency (2 Hz) electrical stimulation, and a control group of placebo transcutaneous acupoint stimulation. The aim of the study is to evaluate the clinical efficacy of transcutaneous acupoint electrical stimulation in treating spasticity during the period after brain damage, exploring ways to improve the effects of spasticity treatment and establishing a clinical protocol for transcutaneous acupoint-electrical stimulation in the treatment of spasticity, in order to minimize the disability rate.

1. **Detailed clinical study**

## 3.1 Study objectives

This study uses a multicenter, clinical, randomized, double-blind, placebo-controlled study (RCT) to evaluate the efficacy of transcutaneous acupoint electrical stimulation (HANS) in relieving muscle spasticity in patients with spasms.

## 3.2 Design of study

### 3.2.1 Source of study subjects

The study aims to recruit appropriate patients from community health centers in Tianjin and its districts. It intends to select patients with limb spasticity who entered these community health centers during the sequelae period following stroke or other brain damage between December 2010 and April 2011.

### 3.2.2 Grouping and randomization of study subjects

The complete randomized grouping will be through random numbers generated by SPSS13.0, allocated by personnel responsible for the randomization scheme. The study population will comprises3 groups: 1) test group of transcutaneous acupoint high frequency (100 Hz) electrical stimulation, 2) test group of transcutaneous acupoint low frequency (2 Hz) electrical stimulation, 3) control group of placebo transcutaneous acupoint stimulation.

To avoid the effects of the researcher’s subject bias on the authenticity of the clinical study results, the randomization will be undertaken via sealed envelopes. The detailed method is as follows: the sequence of the research personnel responsible for recruiting and assessing the subjects is allocated and placed into the coded, sealed, and non-transparent envelopes (the contents of the letter should not be readable under a bright light). In order to prevent the disruption of the allocation sequence, the names and DOB of test subjects are written on the envelope, and the sealed envelopes and detailed patient profiles are video-recorded. The carbon paper in the envelope will transmit the name and DOB information onto the allocation card in the envelope. A second researcher will watch the video and confirm that the envelope has remained sealed following the writing of the subject’s name. Only after the enrolled subjects have completed the baseline assessment can the corresponding envelopes be opened when the treatment intervention is allocated.

### 3.2.3 Blinding method and baseline date collection prior study

(1) Double blinding

The people responsible for evaluating the efficacy and collecting the study data are blinded to the grouping, even during the phase of summarizing the data; the statistician who analyses the data is also blinded to the grouping. Per the research plan the researcher, the operator, the evaluator, and the statistician will be blinded.

(2) Baseline data prior to the study start

1) Efficacy index

Primary efficacy index: modified Ashworth spasticity score^10^ at the wrist, thumb, and other fingers

Secondary efficacy indices: modified Ashworth spasticity score at the knee and ankle, Disability Assessment Scale, Global Assessment Scale, Functional Ambulation Classification by Holden, and Modified Barthel Index

2) Demographic index: etiology, gender, age, nationality, and medical history

3) Safety index: at least 4 patients have claimed to have various adverse events during the recorded treatment period

### 3.2.4 Estimation of sample size and number of clinical study centers

(1) Estimation of sample size

The study is divided into 3 groups; based on the previous research on this subject: the probability of type 1 error is considered to be less than 0.05 (unilateral), with a power of 90%. The sample size has been estimated as following:

A．Test group of transcutaneous electrical stimulation frequency (100 Hz): Ashworth score in the previous 3-week treatment is 3.0 ± 0.4.^7^

B. Test group of transcutaneous electrical stimulation frequency (2 Hz): Ashworth score in the previous 3-week treatment is 3.3 ± 0.3.^7^

C. Control group of placebo transcutaneous acupoint stimulation-recorded modified Ashworth score prior to the treatment is 3.4 ± 0.2.

Several means of the sample are used to compare sample size, calculated as follows:


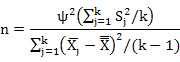


In the equation,
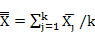
, k is the number of group，
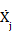
, S_j_ are the mean and standard deviation in group J, ψ is based on α and β, ν_1_=k-1, and ν_2_=k(n-1)ψ value table. Calculating n_(1)_ as ν=∞, calculating n_(2)_ as ν_2_=k[n_(1)_-1], ……, and so on. The homogeneity of the variance test is determined after obtaining the value of n; if the variance is not consistent, then sample size is estimated based on logarithm, homogeneity of variance test is tested after the estimation; and if the homogeneity of the variance test is not consistent, to F’ testing is performed.

Use α=0.05 (unilateral test), $\beta=0.10$, ν_1_=k-1=3-1=2, ν_2_=∞, and check ψ value table and ψ_0.5,0.10,2,∞_=2.52

therefore,


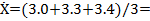
3.23


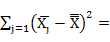
(3.0-3.23)^2^+ (3.3-3.23)^2^+(3.4-3.23)^2^=0.0529+0.0049+0.0289


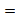
0.0867


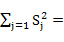
(0.4)^2^+(0.3)^2^+(0.2)^2^=0.16+0.09+0.04=0.29

∴ n=[(2.52)^2^(0.29)/3]/(0.0867/2)= (6.3504×0.29/3)/0.0434=14.14≈15

Based on the calculations above, at least 15 subjects are required for observation in

each group. If ν_2_=3(15-1)=42, check ψ value table, ψ_0.5,0.10,2,42_=2.61; hence,

n=[(2.61)^2^(0.29)/3]/ (0.0867/2)=(6.8121×0.29/3)/0.0434=15.17≈16

In addition, using the homogeneity of variance test via the Bartlett method, the variance is equal (
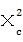
=6.55, *P*>0.005)

With the dropout rate calculated at 15%, the ultimate sample size per group is estimated at 19.

Therefore, the sample size for each group is determined to be 20 per group, with a total of 60 subjects in 3 groups.

Note: The estimated sample size is calculated in accordance with the repeated measurement designs


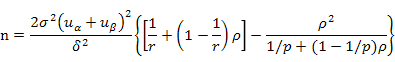


In the equation, p is the number of observations for each subject before intervention, r is the number of observations after intervention; δ=μ_e_-μ_c_, where μ_e_, μ_c_ are the overall means of the test group and control group, respectively, the means estimate the common sample; the range of ρ value is between 0.50-0.75, as ρ=0.65 or ρ=0.70; σ is the overall standard deviation, s is the estimated standard deviation of the sample; α is 0.05, and β is 0.10.

Here, p=1, r=8, δ=3.0-3.4=0.4, ρ=0.65, σ=0.6, α=0.05, β=0.10. Based on these, the sample size for each group from the calculated relevant data of the 100 Hz group and placebo control group is approximately 16 subjects, which is similar to the results calculated above.

(2) Number of clinical study centers

For the purpose of recruiting a sufficient number of subjects within a certain period of time, and thereby achieving a more representative study population, and also for examining the effects of intervention and rare adverse reactions, this clinical study adopts a multicenter design. A total of 6 community centers within Tianjin and all its districts have been selected as branch centers of the study. Meanwhile, the relatively dispersed geographical locations can be used to reflect differences between the regions, social economic status, and lifestyles.

### 3.2.5 Selection of study subjects

(1) Inclusion criteria

Enrolled patients for this clinical study should meet all of the following criteria:

- Aged between 18 and 85 years;
- The combined medical history, cranial CT, or MRI examination shows a diagnosis of cerebral bleeding, cerebral infarction, or cranial trauma of more than 3 months duration;
- Patients present with increased tone of the wrist flexor muscle, with a muscle tone ≥ Grade 3, when 0 is normal power, and 4 is limb stiffness;
- Patients present with difficulties in activities related to personal hygiene and dressing, wrist pain, or abnormal positions, are classified as ≥2 points in the Disability Assessment Score, where 0 is no disability and 3 is the presence of severe disability;^16^
- Patients themselves are able to provide the informed consent or their family understands and agrees to sign the informed consent.

(2) Exclusion criteria

To participate in this clinical study, patients should not have any of the following conditions:

- Presence of severe contractions at the joint of the spastic limb (passive movement range at the joints ≤10°);
- Surgery, including limb-lengthening operations of the Achilles tendon, Achilles tendon transplant, or nerve transection, prior to enrollment in the study;
- Botulinum toxin injection within 4 months prior to study enrollment
  (b) Local neuromuscular blockage on an affected limb within 2 weeks prior to study enrollment
  (c) Oral antispastic therapy;
- Severe atrophic or infected limb muscles;
- Pregnant or breast-feeding (for female subjects), severely impaired liver function, or a pacemaker fitted.

(3) Elimination and withdrawal criteria

- All patients in the study who do not meet the inclusion criteria should be eliminated from the study.
- Patients not treated according to the study protocol or who have incomplete medical information that affects the evaluation of the efficacy or safety of the procedure should be eliminated.
- Patients with poor treatment compliance and patients who withdraw voluntarily.
- Patients who use prohibited concomitant treatment or who voluntarily replace therapy during the study.
- Patients who display severe adverse events or complications, and are therefore not suitable for continuing treatment so the study is terminated.

(4) Management of eliminated and dropped-out subjects

- When a subject drops out, the physician in charge should make a home visit, make an appointment over the phone, or via mail to contact the patient and find out the reason for dropping out, as far as possible. The last treatment time should be recorded and the assessment items should be completed if possible.
- For patients who withdraw from the study due to adverse reactions or ineffective treatment, the physician in charge should manage these subjects as appropriate, based on the individual situation.
- Once the random number has been allocated, the patient immediately becomes a subject for observation, no matter what the diagnosis is, or whether the treatment is complete.
- “Completing the study” part in the case report form (CRF) should be filled in within the correct time period.
- All subjects who are eliminated or withdraw will be included in the intention-to-treat analysis after the completion of the study.

(5) Discontinuing study cases

- Subjects who present with severe adverse events during the study should not continue the study.
- Subjects who become critically ill and require emergency intervention.
- Subjects who wish to withdraw from the study.
- Subjects who are uncooperative, poorly compliant with treatment, and who do not respond to the repeated explanations of the physician.
- The researcher clearly documents the reasons and time of withdrawal, the data of those subjects who are more than halfway through treatment should be included in the efficacy statistics.

### 3.2.6 Treatment regimen

All subjects should receive conventional treatment, such as education on spasm rehabilitation, diet control, and appropriate rehabilitation. This clinical study does not limit a patient’s use of medication that do not affect the treatment outcome of the study. However, patients are asked to document the drugs they have used.

The method of transcutaneous acupoint electrical stimulation is described below:

- Transcutaneous acupoint electrical stimulation of 100 Hz: The patient’s limb is selected randomly to place a circular surface-stimulating electrode (about 24 mm in diameter) at the “Hegu” and “Thenar” points, forming a loop; in addition, a body surface electrode is also placed at ipsilateral “Zusanli” and “Chengshan” points, forming another loop. These 2 pairs of electrodes are connected with the HAN’S acupoint nerve stimulator respectively (HANS-100A; Nanjin Jisheng Pharmaceutical Technology Co., Ltd.). Electrical stimulation is applied to the 2 loops, at a frequency of 100 Hz and a pulse width of 0.2 ms, with the amplitude gradually increasing from 0 mA to the maximum that could be tolerated (the maximum output intensity of the machine is 50 mA). At the maximum tolerated level, generally with an intensity of 20-40 mA, rhythmic contractions of the hand and calf muscles are visible, and there is no subjective discomfort or pain. The treatment is given once daily for 30 minutes per session, 5 days per week.
- Transcutaneous acupoint electrical stimulation of 2 Hz, in conjunction with conventional treatment: this group of patients is given 2 Hz of transcutaneous acupoint-electrical stimulation. The patient’s limb is selected randomly to place a circular surface-stimulating electrode (about 24 mm in diameter) at the “Hegu” and “Thenar” points, forming a loop; in addition, a body surface electrode is placed at the ipsilateral “Zusanli” and “Chengshan” points, forming another loop. These 2 pairs of electrodes are connected with the HAN’S acupoint nerve stimulator, respectively. Electrical stimulation is applied to the 2 loops, at a frequency of 2 Hz and a pulse width of 0.2 ms, with the amplitude gradually increasing from 0 mA to the maximum that could be tolerated (the maximum output intensity of the machine is 50 mA). At the maximum tolerated level, generally with an intensity of 20-40 mA, rhythmic contractions of the hand and calf muscles are visible, and there is no subjective discomfort or pain. The treatment is given once daily for 30 minutes per session, 5 days per week.
- Control group of transcutaneous acupoint placebo stimulation, in conjunction with conventional treatment: this group of patients receives the same acupoint surface stimulation electrode as the treatment groups, once daily, for 30 minutes per treatment. The patients can see the electrical stimulator working, but the intensity of the electrical stimulator is set at 0 mA.

Based on the early-stage study, this clinical treatment should be carried out for 4 weeks, followed by 8 weeks’ follow-up.

### 3.2.7 Assessment system

During the period of treatment and post-treatment follow-up, the assessor who had received training will assess the patient’s relevant index once every 2 weeks, and fill out the CRF. The assessment system is detailed below:

(1) Efficacy index

The study mainly assesses 3 clinical efficacy indices, namely the primary index (the modified Ashworth Spasticity scale, Table 1) and the secondary indices (Disability Assessment Scale [Table 2], Global Assessment [Table 3], 10-meter walking speed [Table 4], and Modified Barthel Index [Table 5]).

**Table 1. Modified Ashworth Spasm Scale**

| Score | Criteria |
| --- | --- |
| 0 | No increase in muscle tone，no resistance at the end of the range of movement (ROM) on the affected limb |
| 1 | Slight increase in muscle tone, slight resistance at the end of the ROM on the affected limb |
| 1^+^ | Slight increase in muscle tone, manifested by a “catch” when the affected limb is in the first ½ ROM, slight resistance in the second ½ ROM. |
| 2 | Mild increase in muscle tone, resistance through most of the ROM on the affected limb, but easily moved. |
| 3 | Moderate increase in muscle tone, resistance throughout the ROM on the affected limb, with difficulty in movement. |
| 4 | Severe increase in muscle tone, stiffness of the affected limb, with considerable resistance, difficulty with passive movement. |

*ROM, range of movement.

**Table 2 Disability Assessment Scale**

| Range of function | Degree of self-function impairment | | | |
| --- | --- | --- | --- | --- |
|  | No disability | Slight disability | Moderate disability | Severe disability |
| Personal hygiene | 0 □ | 1 □ | 2 □ | 3 □ |
| Dressing | 0 □ | 1 □ | 2 □ | 3 □ |
| Limb posture | 0 □ | 1 □ | 2 □ | 3 □ |
| Pain | 0 □ | 1 □ | 2 □ | 3 □ |

The main goal of treatment: Both the patient and the nursing staff should select the functional category they would most like to improve (each should select one of the 4 functional categories).

【Assessment and notices】

The assessor interviews each patient to gauge the severity of the functional impairment in personal hygiene, dressing, limb posture, and pain. The scale score is assessed based on the following: 0, no disability; 1, mild disability (obvious, but not severe enough to affect normal functioning); 2, moderate disability (requires self-efforts and/or assistance from others to achieve normal function); 3, severe disability (restricted normal function).

The 4 functional categories above are assessed based on the following guidelines:

**Personal hygiene:** The patient is assessed in terms of degree of palm wasting, ulcers and/or infection, hygiene of palms and nails, difficulty in cleaning, difficulty in nail trimming, and the degree of disturbance related to hygiene in the patient’s daily living.

**Dressing:** Difficulty in dressing (e.g., indoor clothes, coats, jackets, and gloves) and the degree of disturbance related to dressing in a patient’s daily living are assessed.

**Limb posture:** The ability of the affected limb to make and maintain certain postures (applying make-up, brushing hair, or waving) is assessed.

**Pain:** The pain score related to increased muscle movements in the upper limbs, and the impact of limb pain and discomfort in a patient’s daily living is assessed.

**Table 3. Global Assessment Scale**

| 【Item and assessment criteria】Global assessment scale only has one item, i.e., Conditions, divided into100 grades. The assessment should not only consider the severity of various mental illnesses, but also consider the level of social function. The lower the score, the worse the condition. Score 1-10 is the most serious, meaning the most dangerous, severely affected patients who need a diurnal guardian, or a patient who is independent of daily activities. Score 91-100 is the mildest, meaning the mental state is normal with extremely good adaptation ability, no personality defect, and can deal with all kinds of difficulties. | |
| --- | --- |
| 91-100 | Superior functioning in a wide range of activities, life’s problems never seem to get out of hand, is sought out by others because of his or her many positive qualities. No symptoms. |
| 81-90 | Good functioning in all areas, interested and involved in a wide range of activities, socially effective, generally satisfied with life, at most temporary symptoms, no more than everyday problems or concerns that seem to get out of hand. |
| 71-80 | At most, mild impairment with functions, various degrees of everyday concerns or problems, sometimes they seem to get out of hand. With or without mild symptoms |
| 61-70 | Some mild symptoms (e.g., depressed mood and mild insomnia) or some difficulty in social, occupational, or school functioning (e.g., occasional truancy, or theft within the household), but generally functioning pretty well, has some meaningful inter-personal relationships. Most people without professional training cannot tell the person is sick. |
| 5l-60 | Moderate symptoms or moderate difficulty general functioning *(*e.g., few friends, conflicts, flat affect, depressed mood, pathological self-doubt, euphoric mood, spout eloquent speeches, moderate anti-social behavior, etc.) |
| 41-50 | Serious symptoms or impairment in functioning. Most clinicians think patients require treatment or attention (e.g., suicidal ideation or suicidal state, severe obsessive symptoms or presentations, frequent anxiety attacks, severe anti-social behavior, compulsive alcoholism, certain moderate manic symptoms. |
| 31-40 | Severe impairment in several fields, such as working, family relationship, judgment, thinking, mental state (e.g., suppressed woman avoiding friends, neglecting family and is unable to work), reality testing (e.g., hallucination and illusion), or communication (e.g., speech is at times illogical, obscure, or irrelevant); or suicidal behaviors. |
| 21-30 | Inability to function in almost all areas (e.g., stays in bed all day), behavior is considerably influenced by delusions or hallucinations or serious impairment, in communication or judgment (e.g., sometimes incoherent, no responses) or judgment (highly inappropriate act). |
| 11-20 | Requires some monitoring and management to prevent from some danger of hurting self or others; inability to maintain minimal personal hygiene (e.g., repeated suicidal behaviors, frequent violent behaviors, manic excitement, smears feces); or severe impairment in communications (e.g*.*, largely incoherent or mute). |
| 1-10 | Persistent danger of severely hurting self or others that requires consistent, days of monitoring management. Or severe suicidal behaviors with clear expectation of death. |

Total points:

【Assessment and notices】

1．When a patient’s clinical condition meets several assessment criteria at different levels, the most serious degree should be taken into account. If a patient has mild depression (score assessed as 61-70) and fragments of delusion (reality testing) (score assessed as 31-40), the score recorded should be the latter, i.e., 31-40.

2．Firstly, the grade range for the condition, e.g., 31-40 or 41-50, should be assessed. Then, based on the specific conditions, the severity within the category should be assessed specifically, e.g., if the condition is within the main category 31-40, and is more severe within that range, then the score should be assessed at 32 or 33.

**Table 4. Holden Classification of Walking Ability**

| Score |  | Criteria |
| --- | --- | --- |
| 0 | □ | No walking ability, patient is unable to walk, or requires 2 people to assist in walking |
| 1 | □ | Requires considerably constant assistance, requires 1 person to constantly assist in walking to keep balance. |
| 2 | □ | Requires a little assistance, able to walk with poor balance, not safe, requires 1 person to assist in body contact intermittently to keep balance and ensure safety. |
| 3 | □ | Requires care or verbal guidance, able to walk, but not safe enough, requires 1 person to supervise or guide verbally, without body contact, |
| 4 | □ | Completely independent on the flat ground, can walk independently on the flat ground, but has difficulties in walking on up and down a slope or uneven ground or up or down the stairs, requires help or care from others. |
| 5 | □ | Completely independent, can walk independently anywhere. |

**Table 5. Modified Barthel index**

| ADL item | Independent | Supervising and tips | Slightly independent | Attempt but not safe | Needs help to complete |
| --- | --- | --- | --- | --- | --- |
| Food | 10 | 8 | 5 | 2 | 0 |
| Shower | 5 | 4 | 3 | 1 | 0 |
| Trimming | 5 | 4 | 3 | 1 | 0 |
| Dressing | 10 | 8 | 5 | 2 | 0 |
| Large bowel control | 10 | 8 | 5 | 2 | 0 |
| Small bowel control | 10 | 8 | 5 | 2 | 0 |
| Toilets | 10 | 8 | 5 | 2 | 0 |
| Transfer from bed to chair | 15 | 12 | 8 | 3 | 0 |
| Walk | 15 | 12 | 8 | 3 | 0 |
| Up and down stairs | 10 | 8 | 5 | 2 | 0 |

(2) Safety assessment

For adverse reactions occurring in different treatment regimen in the study, statistical comparisons will be performed, making a safety assessment for the different treatment groups.

For the convenience of observation of each index of the patient by the researchers, the research group will create a CRF table, including each index, observation time, documentation of adverse events, and safety assessment. The researchers will fill in the relevant information, according to the requirements of the CRF table, promptly and accurately.

### 3.2.8 Quality control

(1) Establishing a quality control system

The research group has established 2 levels of quality inspection. The first is an inspection of quality control by the quality inspector appointed by the clinical research center responsible for establishing the quality control list. The quality inspector, based on the quality inspection list, inspects all the records of the original research data, data reports, and adverse events; the central person in charge should actively take corresponding measures toward the quality problems and manage these accordingly and promptly. The second level of control is quality monitoring: the lead researcher is responsible for directly appointing a qualified inspector and planning the number of inspectors and the times of inspection based on a clinical experimental study plan and the speed of the research.

The quality inspector specifically supervises the implementation of, for example, the study scheme and actual flow chart controlled by the clinical physician, to confirm that all records of research data, reports, and case report forms are factual, accurate, and complete, as well as consistent with the original data.

(2) Quality control

- For the characteristics and difficulties of the clinical study, overall analysis of possible confounding factors should be performed, to reduce the biases of research outcomes.
- The complexity of the scheme of clinical treatment should be fully recognized, and the protocols of various treatment operation strictly followed; the clinical treatment personnel are all postgraduates with a certificate of medical practice or clinical acupuncture physicians.
- The randomization plan for group therapy should be strictly carried out.
- The filling of the assessment forms, assessment method, and assessment time by researchers familiar with the various assessment tools should be standardized and calibrated. The clinical assessor strictly abides by this principle to make the clinical assessments.
- The completion instructions for CRF tables should be strictly followed, and the detailed information recorded for the observed subject. Data entry should be careful and complete and corresponding inspection reports should be attached, with no changes made arbitrarily to the original data. If there are any changes, details of the reason(s) for these must be specified and signed by the person who has made the changes.
- Abnormal criteria for laboratory investigations should be based on the normal reference range of the laboratory performing the tests.
- Drugs and devices used in the study should be purchased as a single lot, to assure the reliability of the study conclusions.
- The implementation of each scheme should ensure the patient’s compliance.

### 3.2.9 Management of adverse events

When adverse events occur, the researchers should take such measures necessary based on the patient’s condition and decide whether or not to stop the study. Any laboratory abnormalities should be tracked until these have returned to normal.

No matter whether or not the adverse reactions or events are related to the study treatment, the research personnel should record details of these events thoroughly, including a description of the adverse events and severe adverse events, onset, time of termination, duration (counted in days or hours), severity and frequency of occurrence, their management, treatment volume, course, and justification, and the treatment outcome. The causal relationship between the adverse events and the study treatment should be analyzed. Follow-up of the adverse events and severe adverse events should be continued for as long as necessary, until the events have resolved. All clinical data of adverse events related, such as laboratory results and ECG, shall be recorded in the original file.

### 3.2.10 Ethical clearance from the committee and registration of clinical study

The clinical study scheme has been agreed by the main research personnel, following the clearance from the ethical committee, based on the Helsinki declaration, and after referring to the requirements for a pharmaceutical clinical study and for an informed consent form. In the event of damage related to the clinical study, the subjects should be able to obtain appropriate compensation.

Following the clearance from the ethical committee, the study has been registered with the China clinical study registration center as a clinical study registration platform before the study begins.

### 3.2.11 Statistical analysis

(1) Statistical analysis plan and statistics software

Professional statisticians, in addition to the lead researcher, are responsible for the statistical analysis. Based on the clinical study scheme, the statistical analysis plan has been established. The SPSS13.0 statistical analysis system will be used as the statistical software.

(2) Content and method of statistical analysis

1) The main analysis includes:

- Distribution of cases: data collected for each subject, distribution of subjects at each center, total number of withdrawals, number of subjects terminating the study, and the reason for termination shall be listed in detail.
- Comparison of balance: a comparison of demographic data and other basic indices, analyzing the comparability of the 2 groups.
- Analysis of efficacy: calculation of the efficacy scores of patients at randomization, during the treatment course, and 2 months after treatment, using the differences between the mean and standard deviations with the baseline scores during the treatment to describe it. If the main index is to count data in a normal distribution, a single factor analysis of variance or repeated measurement analysis for statistical tests or the Pearson correlation analysis will be used. If the main index is a skewed distribution or is a measurement data, then the rank sum test or Spearman rank correlation for statistical analysis will be used.
- Safety analysis: firstly based on the requirement for the adverse drug reaction relevance, adverse events and adverse reactions will be listed and described for each group (including various cases of adverse events and their incidence), along with the reason for each and their explanations. If a comparison is needed, the rank sum test for the statistical analysis for adverse reactions will be used.

2) Analytical method: all data is expressed in $\overline{X}\pm SD$ If there are count data in a normal distribution, the single factor analysis of variance or repeated measurement analysis for statistical tests or the Pearson correlation analysis will be used. If the main index is a skewed distribution, or is a measurement data, the rank sum test or Spearman rank correlation for statistical analysis will be used.

P<0.05 is considered to indicate statistical difference.

1. **Study design**


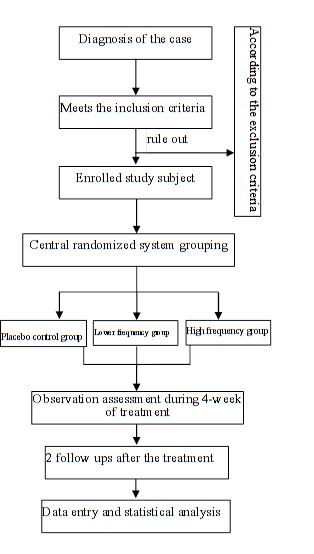


1. **References**

Lance JW. The control of muscle tone, reflexes and movement: Robert Wartenberg lecture. Neurology. 1980;30(12):1303-13.

^2^ van Kuijk AA, Hendricks HT, Pasman JW, et al. Are clinical characteristics associated with upper-extremity hypertonia in severe ischaemic supratentorial stroke? J Rehabil Med. 2007;39(1):33-7.

^3^ Verplancke D, Snape S, Salisbury CF, et al. A randomized controlled trial of botulinum toxin on lower limb spasticity following acute acquired severe brain injury. Clin Rehabil. 2005;19(2):117-25.

^4^ 励建安，朱晓军，敖丽娟等. 肉毒毒素治疗成人肢体肌痉挛：中国指南（2010）. 中国康复医学杂志，2010，25（6）：595-620

^5^ Kasdon DI, Lathi ES. A prospective study of radio-frequency rhizotomy in the treatment of posttraumatic spasticity. Neurosurgery. 1984;15(4):526-9.

^6^ Kamen L, Henney HR 3rd, Runyan JD. A practical overview of tizanidine use for spasticity secondary to multiple sclerosis, stroke, and spinal cord injury. Curr Med Res Opin. 2008;24(2):425-39.

^7^ Rekand T. Clinical assessment and management of spasticity: a review. Acta Neurol Scand Suppl. 2010;(190):62-6.

^8^ Bajd T, Gregoric M, Vodovnik L, et al. Electrical stimulation in treating spasticity resulting from spinal cord injury. Arch Phys Med Rehabil. 1985;66(8):515-7.

^9^ 汪家琮，严尚诚，韩济生等. 穴位经皮神经电刺激（HANS）治疗脊髓损伤引起的痉挛. 中国康复理论与实践，1997，3（3）：111-117

^10^ Bohannon RW, Smith MB. Interrater reliability of a modified Ashworth scale of muscle spasticity. Phys Ther. 1987;67(2):206-7.

^1^ Brashear A, Zafonte R, Corcoran M, et al. Inter- and intrarater reliability of the Ashworth Scale and the Disability Assessment Scale in patients with upper-limb poststroke spasticity. Arch Phys Med Rehabil. 2002; 83(10):1349-54.

^2^ Endicott J, Spitzer RL, Fleiss JL, Cohen J. The global assessment scale. A procedure for measuring overall severity of psychiatric disturbance. Arch Gen Psychiatry. 1976;33(6):766-71.

^3^ Viosca E, Martínez JL, Almagro PL, et al. Proposal and validation of a new functional ambulation classification scale for clinical use. Arch Phys Med Rehabil. 2005;86(6):1234-8.

^4^ Shah S, Vanclay F, Cooper B. Improving the sensitivity of the Barthel Index for stroke rehabilitation. Journal of Clin Epidemiol. 1989;42(8):703-9.

^5^ Shah S, Vanclay F, Cooper B. Predicting discharge status at commencement of stroke rehabilitation. Stroke. 1989;20(6):766-9.

^6^ Brashear A, Gordon MF, Elovic E, et al. Intramuscular injection of botulinum toxin for the treatment of wrist and finger spasticity after a stroke. N Engl J Med. 2002;347(6):395-400.

**经皮穴位电刺激治疗肌肉痉挛**

**的随机对照临床研究**

申 请 者： 赵 晔

所在单位： 天津中医药大学针灸学院

通讯地址： 天津市南开区鞍山西道312号

电 话: 13662121309 E-mail: zhaoye@tjutcm.edu.cn

传 真: 022-27485189邮政编码: 300193

起止年限: 2010年12月——2012 年 5月

二○一○年制

**主要成员的业务简历**

| 赵晔，天津中医药大学针灸推拿学专业博士在读，主要从事针灸相关临床试验研究。2002.9——2005.7就读于天津中医药大学中西医结合基础专业，取得医学硕士学位，学位论文题目为“井穴放血及中药对大鼠局灶性脑缺血损伤后神经干细胞再生的影响”；2005.7——2008.7在天津市儿童医院中西医结合科从事临床医疗工作，职务为医师。  韩济生，教授, 博士生导师，中国科学院院士，北京大学神经科学研究所名誉所长。长期从事针刺镇痛原理研究。初步阐明针刺镇痛的神经化学原理，获国家自然科学三等奖（1987）。在此基础上，阐明不同频率的电针刺激可引起不同种类神经肽释放的生理机制和分子机制，获国家自然科学二等奖（1999）。与北京航空航天大学刘亦鸣教授合作研制成功韩氏穴位神经刺激仪（HANS），可进行数字化的电针治疗及跨皮肤神经刺激治疗。2010年8月在第13届国际疼痛大会上应邀做针刺镇痛大会报告。  郭义，教授，博士生导师，天津中医药大学针灸学院院长。主持和承担国家“八五”攀登经络的研究子课题、国家“八五”攻关、“十五”攻关、国家“973”子项目、国家自然科学重点基金、国家自然科学基金、教育部霍英东青年教师基金及部、市级课题50余项，主持和参与制定国家针灸标准5项，已发布。 |
| --- |

**目录**

[**摘要** 1](#__RefHeading___Toc280025285)

[**1．** **研究背景与意义** 1](#__RefHeading___Toc280025286)

[**2．** **国内外研究现状及课题组前期工作基础** 2](#__RefHeading___Toc280025287)

[**3．** **临床试验具体内容** 2](#__RefHeading___Toc280025288)

[**3.1 研究目的** 2](#__RefHeading___Toc280025289)

[**3.2 试验设计** 3](#__RefHeading___Toc280025290)

[**3.2.1 研究对象的来源** 3](#__RefHeading___Toc280025291)

[**3.2.2 研究对象的分组及随机化隐匿** 3](#__RefHeading___Toc280025292)

[**3.2.3 盲法及验前基线数据采集** 3](#__RefHeading___Toc280025293)

[**3.2.4 样本含量估计及临床试验中心数量** 4](#__RefHeading___Toc280025294)

[**3.2.5 受试对象的确定** 6](#__RefHeading___Toc280025295)

[**3.2.6 治疗方案** 7](#__RefHeading___Toc280025296)

[**3.2.7 评价体系** 8](#__RefHeading___Toc280025297)

[**3.2.8 质量控制** 11](#__RefHeading___Toc280025298)

[**3.2.9 不良事件的处理** 12](#__RefHeading___Toc280025299)

[**3.2.10 伦理学委员会审批与临床试验的注册申请** 13](#__RefHeading___Toc280025300)

[**3.2.11 统计分析** 13](#__RefHeading___Toc280025301)

[**4. 技术路线图** 14](#__RefHeading___Toc280025302)

[**参考文献** 15](#__RefHeading___Toc280025303)

**摘要**

痉挛是大脑和脊髓水平的上运动神经元损伤的临床体征之一。据统计大约有20%——40%的脑卒中患者在发病后三个月内，60%的重度多发性硬化患者以及75%的重度创伤性脑损伤后身体残疾的患者会罹患亟待治疗的肌痉挛。及早治疗严重的肌痉挛可避免继发功能损害及活动能力的丧失。现有的手术治疗在解除痉挛的同时也损伤了正常功能，一般不作为首选治疗方法；药物治疗对缓解痉挛具有一定效果，但这些药物往往作用短暂，在解痉的同时也减弱了肌力，加之药物的大剂量使用易导致患者出现镇静状态、呼吸抑制、幻觉或心电图出现QT间期延长等严重副作用，限制了它们的广泛应用。我们根据前期工作基础，拟进行多中心大样本的随机双盲平行对照临床试验，以评估经皮穴位电刺激对于治疗脑血管意外和脑创伤患者后遗症期肢体痉挛的临床有效性和安全性，为在临床中推广本疗法提供客观依据。

1. **研究背景与意义**

痉挛(spasticity)是“上运动神经元综合征患者肢体被动运动阻力的速度依赖性增强”[[1]](#endnote-2)，为上运动神经元损伤的临床体征之一。上运动神经元位于大脑和脊髓中，其损伤通常是由于脑中风、多发性硬化、脊髓损伤、脑损伤、脑瘫以及其他大脑或脊髓病变。其临床表现为肌肉张力增高、深肌腱反射活跃甚至亢进，此系缺乏上位中枢的抑制所致。据估计大约有20%-40%的脑卒中患者在发病后三个月内[[2]](#endnote-3)，60%的重度多发性硬化患者以及75%的重度创伤性脑损伤后身体残疾的患者会发生需要治疗的肌痉挛[[3]](#endnote-4)。

痉挛的表现在不同病人之间差异很大，一般程度的痉挛也可产生有益的作用。在痉挛状态下，肌肉不会发生萎缩，可有效预防深静脉血栓、肢体水肿，骨质疏松。对截瘫患者而言，一定程度的痉挛可维持坐姿、转移、站立甚至行走，对预防压疮也有帮助。当痉挛影响患者的日常生活能力、步态、睡眠、个人卫生；或当痉挛引起严重疼痛、导致挛缩时；亦或是痉挛所致的尴尬使病人隔离社会，慢性疼痛导致抑郁等心理改变时，均要求予以积极处理。严重的肌痉挛如果不予治疗，就会发生恶性循环，受累肌群没有力量对抗痉挛性张力障碍所致收缩，结果造成肢体姿势异常，从而导致软组织缩短，收缩的肌肉发生进一步的生物力学变化。这些变化进而可阻碍肌肉的伸长，进一步加重张力障碍。及早治疗肌痉挛可避免继发适应不良、功能损害，以及避免丧失活动和参加活动的能力[[4]](#endnote-5)。

1. **国内外研究现状及课题组前期工作基础**

目前，国内外对于痉挛主要有以下几种治疗方法。采用手术切断神经、肌腱延长术以及肌腱移植术在解除痉挛的同时也损伤了正常功能，是有创且不可逆的，一般不作为首选治疗方法[[5]](#endnote-6)。巴氯芬、替扎尼定、苯二氮卓类等药物对缓解痉挛具有一定效果，但这些药物往往作用短暂，在解痉的同时也减弱了肌力，对病人整体神经功能改善不大，加之大剂量使用这类药物易导致患者出现镇静状态、呼吸障碍、幻觉、心电图出现QT间期延长等严重副作用，也限制了它们的广泛应用[[6]](#endnote-7)。注射肉毒毒素是目前较为流行的治疗手段，但由于其主要被用于缓解局部小范围的痉挛，也限制了它的临床应用[[7]](#endnote-8)。经皮神经电刺激通过置于皮肤表面的电极刺激神经，与以上方法相比，具有简便、无损害、无副作用的特点[[8]](#endnote-9)。在我们前期临床试验中，已初步证明经皮穴位电刺激在治疗脊髓损伤引起的痉挛中，高频刺激（100Hz）具有明显的即时和持久解痉效果，且与腰部和骶部刺激相比，穴位刺激的疗效持续时间最为持久[[9]](#endnote-10)。

基于前期经皮穴位电刺激治疗脊髓损伤引起痉挛的工作基础上，本课题拟通过多中心大样本随机对照双盲临床试验（RCT），观察在脑中风和脑创伤后遗症期出现肢体痉挛的患者。通过改良的Ashworth评分结果，将入选患者随机分为：经皮穴位高频（100Hz）电刺激试验组、经皮穴位低频（2Hz）电刺激试验组、经皮穴位假刺激对照组。本研究旨在客观、科学的评价经皮穴位电刺激治疗脑损伤后遗症期痉挛的临床疗效，寻找提高痉挛治疗效果的治疗方法，建立经皮穴位电刺激治疗痉挛的临床规范，最大可能地降低致残率。

1. **临床试验具体内容**

**3.1 研究目的**

本试验采用多中心临床随机双盲对照试验(RCT)评价经皮穴位电刺激(HANS)对于缓解痉挛患者肌肉痉挛程度的治疗效应。

**3.2 试验设计**

**3.2.1 研究对象的来源**

本研究主要在天津市内及各区县的社区卫生中心选取相应的患者。拟选择在2010.12——2011.4期间进入各社区卫生中心的脑中风和脑创伤后遗症期肢体痉挛的患者。

**3.2.2 研究对象的分组及随机化隐匿**

根据SPSS13.0产生的随机数字进行完全随机化分组，由专人负责随机方案的分配。本研究设立了三个组别：（1）经皮穴位高频（100Hz）电刺激试验组；（2）经皮穴位低频（2Hz）电刺激试验组；（3）经皮穴位假刺激对照组。

为防止研究者的主观偏倚影响临床研究结果的真实性，随机化的隐匿采用密闭信封法。具体方法如下：对负责招募和评估受试者的研究人员隐藏分配顺序，分配序列装于按顺序编号的密封的不透明的信封中（应严格保证在强光下仍不能看到信瓤的内容）。为防止分配序列被打乱，把受试者的姓名和出生日期写在信封上，并将密封信封和受试者的详细信息记录在录影中。信封内的复写纸将这些信息转写到信封内的分配卡上，然后再由第二位研究人员观看录影，确认写上受试者姓名后的信封依然密封。只用在招募的受试者完成全部基线评估后，才打开相应的信封，这时候再分配干预措施。

**3.2.3 盲法及验前基线数据采集**

（1）双盲法

为疗效评价者和实验数据统计者受盲，即不知分组情况的疗效评价人员对病人的治疗效果进行评价；在资料总结阶段，不知分组情况的统计者进行数据的统计分析。本课题实行研究者、操作者、评价者、统计者四者分离。

（2）验前基线数据

1）疗效指标——

主要疗效指标：对腕、大拇指、其余四指行改良的Ashworth痉挛评分[[10]](#endnote-11)

次要疗效指标：对膝、踝关节行改良的Ashworth痉挛评分；残疾评价量表（Disability Assessment Scale）[[11]](#endnote-12)；大体评价量表（Global Assessment Scale）[[12]](#endnote-13)；Holden步行功能分级（Functional Ambulation Classification）[[13]](#endnote-14)；改良的Barthel指数（Modified Barthel Index）[[14]](#endnote-15)-[[15]](#endnote-16)。

2）人口学指标——病因、性别、年龄、民族、病史；

3）安全性指标——记录治疗期间至少有四名患者声明出现的各种不良反应（Adverse Events）。

**3.2.4 样本含量估计及临床试验中心数量**

（1）样本含量估计

本试验共分为三组，根据以往本课题组研究基础以及参考相关文献，控制第一类错误的概率小于0.05（单侧），检验效能为90%，估计各组样本含量，具体过程如下。

A．经皮穴位高频（100Hz）电刺激试验组——既往试验中治疗三周Ashworth评分为3.0±0.4Error: Reference source not found。

B. 经皮穴位低频（2Hz）电刺激试验组——既往试验中治疗三周Ashworth评分为3.3±0.3Error: Reference source not found。

C.经皮穴位假刺激对照组——记载的治疗前改良的Ashworth评分为3.4±0.2。

此处采用多个样本均数比较的样本含量估计公式如下所示：


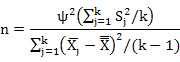


式中
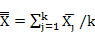
，k为组数，
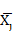
、S_j_为第j组的均数及标准差，ψ值以α、β、ν_1_=k-1、ν_2_=k(n-1)查ψ值表。求n_(1)_时以ν=∞，求n_(2)_时以ν_2_=k[n_(1)_-1]，……，余防此。求出n后再作方差齐性检验，若方差不齐时以对数值再估算样本含量，估算后再作方差齐性检验，若方差不齐则建议试验后作F’检验。

以（单侧检验），，ν_1_=k-1=3-1=2，ν_2_=∞，查ψ值表且得ψ_0.5,0.10,2,∞_=2.52，则


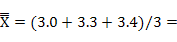
3.23


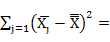
(3.0-3.23)^2^+ (3.3-3.23)^2^+ (3.4-3.23)^2^=0.0529+0.0049+0.0289


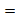
0.0867


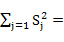
(0.4)^2^+ (0.3)^2^+ (0.2)^2^=0.16+0.09+0.04=0.29

∴ n=[(2.52)^2^(0.29)/3]/(0.0867/2)= (6.3504×0.29/3)/0.0434=14.14≈15

经上述计算，每组至少需要观察15例。若再以ν_2_=3(15-1)=42，查ψ值表得ψ_0.5,0.10,2,42_=2.61，则

n=[(2.61)^2^(0.29)/3]/ (0.0867/2)=(6.8121×0.29/3)/0.0434=15.17≈16

此外，经Bartlett法进行方差齐性检验，得出方差相等（
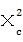
=6.55，*P*>0.005）。

按照脱失率为15%计算，估计最终每组样本量为：19人

因此，拟设计每组样本含量为20人，三组共计60人。

注：按照重复测量设计进行样本量估算


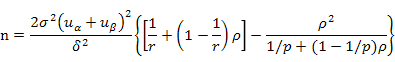


式中p为每个受试者处理前观测次数，r为处理后观测次数；δ=μ_e_-μ_c_，μ_e_、μ_c_分别为试验组和对照组处理后的总体均数，常用样本均数估计；ρ取值范围常在0.50~0.75之间，按照ρ=0.65或ρ=0.70；σ是总体标准差，以样本标准差s估计；α取0.05，β取0.10。

此处，p=1，r=8，δ=3.0-3.4=0.4，ρ=0.65，σ=0.6，α=0.05，β=0.10。由此计算100Hz组与安慰剂对照组的相关数据得出每组样本量近似为16人，与上面的算法所得结果相同。

（2）临床试验中心数量

为在一定时期内征集到足够数量的受试者，并能够真实反映干预措施的效果、少见的不良反应、选择更具代表性的样本，本研究采用多中心临床试验模式。拟定选取天津市内及各区县的社区中心共6家作为本研究的临床分中心。并且，所处的地理位置相对分散，有利于反映不同地区、不同社会经济状况和生活方式的代表性差异。

**3.2.5 受试对象的确定**

（1）纳入标准

入选本临床试验研究的患者同时必须满足下述标准：

- 年龄在18周岁到85周岁之间；
- 结合病史、头CT或MRI检查确诊脑出血、脑梗塞或脑创伤发生3个月以上；
- 患肢腕屈肌出现肌张力升高，经改良Ashworth量表评分肌张力≥3级Error: Reference source not found，其中0表示正常肌力，4表示患肢僵硬；
- 患者存在保持个人卫生以及穿衣的困难，存在腕关节疼痛或位置异常，经残疾评价量表评分≥2分，其中0分表示无残疾，3分表示存在严重残疾[[16]](#endnote-17)；
- 患者本人有能力理解并签署或是其家属理解并愿意签署知情同意书。

（2）排除标准

有资格进入本临床试验研究的患者不能具有以下任何经诊断存在的健康状况：

- 痉挛肢体的关节已发生严重挛缩（其关节被动活动范围≤10°）；
- 入组前曾接受患肢跟腱延长术、跟腱移植术或手术切除神经治疗；
- 入组前4个月曾接受患肢肉毒毒素注射治疗， 入组前2周接受患肢局部神经肌肉封闭或口服解痉剂治疗；
- 患肢肌肉严重萎缩或感染；
- 孕、产妇，严重的肝肾功能损害或装有心脏起搏器。

（3）剔除和脱落标准

- 凡不符合纳入标准而被误入的病例应予剔除；
- 未按规定治疗或资料不全等影响疗效评价和安全性评价者应予剔除；
- 受试者依从性差，疗程中自行退出者；
- 合并使用本方案禁止使用的治疗方法或自行中途更换治疗方法者；
- 发生严重不良事件或并发症，不宜继续接受治疗而被中止试验的病例。

（4）剔除与脱落病例的处理

- 当受试者脱落后，主管医生应采取登门、预约电话、信件等方式，尽可能与受试者联系询问理由，记录最后一次治疗时间，完成所能完成的评估项目；
- 因不良反应、治疗无效而退出试验的病例，主管医生应根据受试者实际情况采取相应的治疗措施；
- 一旦取得随机号码，即成为试验观察的对象，不管以后诊断如何及治疗是否完整；
- 及时填写病例报告表的“研究完成情况”；
- 所有剔除与脱落病例于试验结束后进行意向性分析。

（5）研究病例的中止

- 研究中出现严重的不良反应，不宜继续参加研究者；
- 研究期间受试者出现其他急重症，需采取紧急措施者；
- 受试者中途提出退出临床研究；
- 患者不合作、不服从治疗，经临床医生反复解释无效；
- 研究者详细记录退出研究的原因及时间，已超过1/2疗程者应进入疗效统计。

**3.2.6 治疗方案**

所有受试者均接受痉挛康复教育，通过饮食控制，适当康复训练等常规治疗。本临床试验不限制患者自行应用的对本治疗研究不产生影响的药物，但要求其记录所应用的相关药物。

①经皮穴位高频电刺激（100Hz）试验组：本组患者在常规治疗基础上，予以经皮穴位100Hz电刺激。在患者患肢随机选取“合谷”穴和“鱼际”穴处各放置一个圆形体表刺激电极（直径约24mm），形成一个回路；另外，在同侧的“足三里”穴和“承山”穴处也放置一个体表刺激电极，形成另一个回路。两对电极分别与韩氏穴位神经刺激仪（HANS-100A型，南京济生医疗科技有限公司）相联。给两回路施加电刺激，频率为100Hz，波宽为0.2ms，幅度从0mA逐渐增加至对大而能耐受的程度（仪器的最大输出强度为50mA）。这时可见手及小腿的肌肉有节律地收缩，主观上无不适感或痛感，一般所用强度为20-40mA。每天治疗一次，每周治疗五天，每次的刺激时间为30分钟；②经皮穴位低频电刺激（2Hz）试验组：本组患者在常规治疗基础上，予以经皮穴位2Hz电刺激。在患者患肢随机选取“合谷”穴和“鱼际”穴处各放置一个圆形体表刺激电极（直径约24mm），形成一个回路；另外，在同侧的“足三里”穴和“承山”穴处也放置一个体表刺激电极，形成另一个回路。两对电极分别与韩氏穴位神经刺激仪相联。给两回路施加电刺激，频率为2Hz，波宽为0.2ms，幅度从0mA逐渐增加至对大而能耐受的程度（仪器的最大输出强度为50mA）。这时可见手及小腿的肌肉有节律地收缩，主观上无不适感或痛感，一般所用强度为20-40mA。每天治疗一次，每周治疗五天，每次的刺激时间为30分钟；③经皮穴位假刺激对照组：本组患者在常规治疗基础上，予以治疗组同样穴位体表刺激电极敷贴，每天一次，每次30分钟，患者可以看到电刺激仪在工作，但电刺激仪的刺激强度设定为0mA。

根据前期工作基础，考虑本次临床治疗拟持续4周，其后随访8周。

**3.2.7 评价体系**

在治疗期间以及治疗后的随访期间，由经过统一培训的评估人对患者的相关指标每两周进行一次评价，填写病例报告表。具体评价体系如下，

（1）疗效指标

主要考察3个临床疗效指标，包括：主要指标——改良Ashworth痉挛量表；次要指标——残疾评价量表（Disability Assessment Scale）；大体评价量表（Global Assessment）；10米步行速度；改良的日常生活活动量表（Modified Barthel Index）。

**表1.** 改良Ashworth痉挛量表

| 等 级 | 标 准 |
| --- | --- |
| 0 | 肌张力不增加，被动活动患侧肢体在整个范围内均无阻力 |
| 1 | 肌张力稍增加，被动活动患侧肢体到终末端时有轻微的阻力 |
| 1^+^ | 肌张力稍增加，被动活动患侧肢体时在前1/2 ROM*中有轻微的“卡住”感觉，后1/2 ROM中有轻微的阻力 |
| 2 | 肌张力轻度增加，被动活动患侧肢体在大部分ROM内均有阻力，但仍可以活动 |
| 3 | 肌张力中度增加，被动活动患侧肢体在整个ROM内均有阻力，活动比较困难 |
| 4 | 肌张力高度增加，患侧肢体僵硬，阻力很大，被动活动十分困难 |

*ROM是指活动范围

**表2. 残疾评价量表**

| 功能范畴 | 自身功能障碍程度 | | | |
| --- | --- | --- | --- | --- |
|  | 没有残疾 | 轻微残疾 | 中度残疾 | 严重残疾 |
| 个人卫生 | 0 □ | 1 □ | 2 □ | 3 □ |
| 穿衣 | 0 □ | 1 □ | 2 □ | 3 □ |
| 肢体姿势 | 0 □ | 1 □ | 2 □ | 3 □ |
| 疼痛 | 0 □ | 1 □ | 2 □ | 3 □ |

主要治疗目标：患者最希望得到 功能范畴的改善（请在四个功能范畴中选择一项填写）；患者的主要护理人员最希望患者得到 功能范畴的改善（请在四个功能范畴中选择一项填写）。

【评分及注意事项】

评估人访问每个患者以评价其若干功能损伤的程度，包括：个人卫生、穿衣、肢体位置和疼痛等情况。根据以下情况进行量表评分：0分表示没有残疾、1分表示轻微残疾（明显但并不严重影响正常功能）、2分表示中等程度的残疾（要通过自我努力和/或他人辅助才能实现正常功能）、3分表示严重残疾（正常功能受限）。

上述四类功能范畴的评估基于以下指南：

**个人卫生**：评估人对患者进行评价，根据：手掌消瘦、溃烂和/或感染的程度；手掌和手指的清洁度；清洁的难易程度；指甲修剪的难易程度；以及在患者日常生活中由与清洁相关的残疾所造成的干扰程度。

**穿衣**：评估人评价患者穿衣（如，衬衣、外套、手套）的难易程度，以及在患者日常生活中由与穿衣相关的残疾所造成的干扰程度。

**肢体姿势**：评估人评价患肢做出并保持某一姿势（如化妆、梳头或敬礼等）的能力。

**疼痛**：评估人评价与上肢肌张力增高相关的疼痛的强度，以及肢体疼痛和不适对患者日常生活的影响。

**表3. 大体评定量表**

| 【项目和评定标准】大体评定量表只有一个项目，即病情概况，分成（1～100）100个等级。评定时不但要考虑各类精神症状严重程度，而且还要考虑社会功能的水平。分数越低，病情愈重。1～10分最重，指那些最危险、最严重、需要昼夜监护者，或者是一切生活均需他人照顾的病人；而91～100分则是最轻的，是指精神状态全然正常，社会适应能力极为良好，毫无人格缺陷，能应付各种困难处境者。 | |
| --- | --- |
| 91～100 | 在各方面都有较高的活动能力。日常生活上的问题，从未有无法处理的情况；由于其热情和正直，别人都愿与之相处，没有症状。 |
| 81～90 | 在所有领域中都能良好活动，兴趣和社交好。一般而言对生活是满意的，至多也只有暂时性的症状发生，“日常的”担忧偶尔无法处理。 |
| 71～80 | 至多也只是活动能力有轻度的损害，有不同程度的“日常的”担忧及问题，有时无法处理。或有或无轻度的症状。 |
| 61～70 | 有一些轻度的症状（例如轻度抑郁或轻度失眠等），或者在几个活动领域中有一些困难，但是一般活动还是相当好的，有一些富有意义的人际关系，大多数未经训练的人不会认为他“有病”。 |
| 5l～60 | 中等严重程度的症状，或者一般的活动有一些困难。例如：没有什么朋友，情感平淡，抑郁心境，病态的自我怀疑，欣快心情及言语滔滔不绝，中等严重的反社会行为等等。 |
| 41～50 | 有严重症状或者活动能力的损害。大多数临床医生认为，病人需要治疗或注意，例如：自杀先占状态或自杀姿态，严重强迫症状或表现，频繁的焦虑发作，严重的反社会行为，强迫性酗酒，肯定的中等度的躁狂症状等。 |
| 31～40 | 在多个领域中有严重损害。诸如工作、家庭关系、判断、思考、心境（例如抑郁的妇女回避朋友，对家属不负责任，不能料理家务）、现实检验（例如幻觉或妄想）或交谈（如讲话总是含糊不清，不合逻辑或文不对题）等领域中有某些损害；或者出现自杀行为。 |
| 21～30 | 几乎在所有领域中都不能正常活动（例如整天卧床不起）或者其行为受到妄想或幻觉的相当程度的影响；或者严重的损害，表现于交谈（如有时前后不连贯或没有回答）或判断（如其行为极为不适合）之中。 |
| 11～20 | 需要某些监督管理，才能防止其自杀或伤人；或不能维持起码的个人卫生（如反复的自杀行为、频繁的暴力表现、躁狂性的激动、把粪便弄得一塌糊涂等）；或者有交谈方面的严重损害（如重度不连贯或缄默）。 |
| 1～10 | 需要好多天持续不断的监督管理，才能防止自伤或伤人；或病人没有任何企图想要维持起码的卫生；或有严重的自杀行为，同时还清楚地表示非死不可。 |

总分=

【评分及注意事项】

1．病人的情况同时符合若干等级的评定标准时，按其最严重的等级评定。如某病人，有极轻度的抑郁，又有片断的妄想，按前者应评为61～70，若按后者（现实检验）应评为31～40，则应按后者评定。
2．先按病情评出其大范围的等级，即是31～40，还是41～50。然后，再根据具体病情，评定在这一等级中偏重还是偏轻，给予具体评分，例如大类是31～40，病情在这一等级中偏重，则应评为32或33分。

**表4. Holden步行功能分级**

| 评 分 |  | 标 准 |
| --- | --- | --- |
| 0 | □ | 无步行能力，患者不能走，或需2人协助才能走 |
| 1 | □ | 需大量持续性帮助，需1人连续不断地搀扶才能行走及保持平衡 |
| 2 | □ | 需少量帮助，能行走但平衡不佳，不安全，需1人在旁给以间断的接触身体的帮助以保持平衡和保证安全 |
| 3 | □ | 需监护或言语指导，能行走，但可不够安全，需1人在旁监护或用言语指导，但不接触身体 |
| 4 | □ | 平地上完全独立，在平地上能独立行走，但在上下斜坡、在不平的地面上行走或上下楼梯仍有困难，需他人帮助或监护 |
| 5 | □ | 完全独立，在任何地方都能独立行走 |

**表5.** **改良Barthel指数**

| ADL项目 | 自理 | 监督提示 | 稍依赖 | 尝试但不安全 | 不能完成 |
| --- | --- | --- | --- | --- | --- |
| 进食 | 10 | 8 | 5 | 2 | 0 |
| 洗澡 | 5 | 4 | 3 | 1 | 0 |
| 修饰 | 5 | 4 | 3 | 1 | 0 |
| 更衣 | 10 | 8 | 5 | 2 | 0 |
| 控制大便 | 10 | 8 | 5 | 2 | 0 |
| 控制小便 | 10 | 8 | 5 | 2 | 0 |
| 用厕 | 10 | 8 | 5 | 2 | 0 |
| 床椅转移 | 15 | 12 | 8 | 3 | 0 |
| 行走 | 15 | 12 | 8 | 3 | 0 |
| 上下楼梯 | 10 | 8 | 5 | 2 | 0 |

（2）安全性指标

就本次研究中不同治疗方案出现的不良反应，进行统计比较，做出不同治疗组安全性评价。

为方便研究人员观察记录患者的各项指标，课题组将各项指标、观察时间点、不良事件记录、安全性评价等内容编制成CRF表（Case Report Form，CRF），研究人员必须按照CRF表的要求及时、准确填写相关信息。

**3.2.8 质量控制**

（1）建立质量控制体系

课题组制定了两级质量检查制度，一级为质量控制检查，由临床研究中心负责人任命质量检查员，并制定质量检查清单，质量检查员根据质量检查清单对其全部研究源数据的记录、数据报告及不良事件等进行检查；中心负责人对存在的质量问题积极采取相应措施进行及时处理；二级为质量监查，本课题负责人直接委派具备一定资格质量监察员。并根据临床试验研究计划和研究速度计划监查员人数和监查时间。质量检查员具体监查实施内容包括对涉及临床医师对试验方案和流程的实际掌握情况，确认所有研究数据的记录与报告以及病例报告表填写的真实、准确与完整，并保证与原始资料一致。

（2）质量控制内容

- 针对临床研究的特点与难度，全面分析可能出现的混杂因素，以减少研究结果的偏倚；
- 充分认识到临床治疗方案研究的复杂性，严格执行各种治疗方法的操作规范；临床治疗人员均为取的执业医师资格证的研究生或者临床针灸主治医师。
- 严格执行随机化方案，进行分组治疗；
- 由熟悉量表的研究者规范量表的填写方式、评分方法及评价时点，并就量表相关条目的询问方式进行统一，临床评价人员严格遵循此原则进行临床评价；
- 严格按照CRF表填写说明填写CRF表，记录观察病例详细资料。数据录入要求认真、完整，并将相应的检查报告附后，原始数据不得随意更改，如有更改应必须详细注明原因，更改者签名；
- 实验室检查的异常判断标准，以检查单位的正常参考范围为准；
- 试验中应用的药物及器械须统一购置，以保证研究结论的可靠性；
- 在各方案的实施中保证患者的依从性。

**3.2.9 不良事件的处理**

当发现不良事件时，研究者可根据病情采取必要的处理措施，直到病情稳定，若化验异常者应追踪至恢复正常，并决定是否中止观察。

无论不良反应或不良事件是否与研究治疗方法有关，参与受试者治疗的研究人员均应详细记录，记录内容包括：不良事件与严重不良事件的详细描述、发生时间、中止时间、持续时间（可以用天数或小时来记录）；发生的严重程度及频率；处理方法的操作、治疗量、疗程及理由，并记录治疗结果；对不良事件与试验治疗方法的因果关系的分析。不良事件与严重不良事件的跟踪情况；有关不良事件的所有临床资料，如化验单、心电图等均应记录在原始文件中。

**3.2.10 伦理学委员会审批与临床试验的注册申请**

临床试实验方案由主要研究人员共同商定，包伦理委员会审批后实施。按照《赫尔辛基宣言》并参照《药品临床研究管理规范》的要求，制定知情同意书。若发生与临床研究有关损害，受试者可得到适当补偿。

取得伦理委员会批准后，研究开始前在权威的临床试验研究注册平台中国临床试验注册中心进行注册。

**3.2.11 统计分析**

（1）统计分析计划书与统计软件

由统计专业人员负责，并与主要研究者，根据临床试验方案共同制定统计分析计划书。统计软件采用SPSS13.0统计分析系统进行统计。

（2）统计分析内容和方法

1）主要分析内容包括：

- 病例分布：各组不同数据集大小，各中心病例分布，总脱落病例，中止及原因详细列表。
- 均衡性比较：比较人口学资料和其他基础值指标，衡量两组的可比性。
- 有效性分析：计算患者入组、治疗期间和治疗后2个月随访时的疗效评分，采用治疗期相对基线的差值的均数、标准差进行描述。主要指标如果是计数资料且呈正态分布，采用单因素方差分析或重复测量的方差分析进行统计检验或是Pearson相关分析，主要指标如果是偏态分布或是计量资料采取秩和检验或Spearman秩相关进行统计分析。
- 安全性分析：首先根据不良反应相关性的要求，列表描述各组的不良事件和不良反应（包括各种不良事件的例数和发生率），列出其原因和解释。若需比较的话可采用秩和检验对不良反应进行统计分析。

2）分析方法：全部数据以表示。如果是计数资料且呈正态分布，采用单因素方差分析或重复测量的方差分析进行统计检验或是Pearson相关分析；主要指标如果是偏态分布或是计量资料采取秩和检验或Spearman秩相关进行统计分析。P<0.05为有统计学差异。

**4. 技术路线图**

病例诊断

根据排除标准

本课题研究对象

中央随机系统分组

高频组

假刺激

治疗4周观察评价

治疗后的2次随访

数据录入、统计分析

排除

符合纳入标准

低频组

图1. 技术路线图

**参考文献**

1. Lance WW. The control of muscle tone, reflexes and movement: Robert Wartenberg lecture. Neurology, 1980, 30: 1303–1313 [↑](#endnote-ref-2)
2. van Kuijk AA, Hendricks HT, Pasman JW, et al.Are clinical characteristics associated with upper-extremity hypertonia in severe ischaemic supratentorial stroke? Journal of rehabilitation medicine, 2007, 39 (1): 33-37 [↑](#endnote-ref-3)
3. Verplancke D, Snape S, Salisbury CF, et al. A randomized controlled trial of botulinum toxin on lower limb spasticity following acute acquired severe brain injury. Clinical rehabilitation, 2005, 19 (2): 117-125 [↑](#endnote-ref-4)
4. 励建安，朱晓军，敖丽娟等. 肉毒毒素治疗成人肢体肌痉挛：中国指南（2010）. 中国康复医学杂志，2010，25（6）：595-620 [↑](#endnote-ref-5)
5. Kasdon DI, et al. A prospective study of radio-frequency rhizotomy in the treatment of post-traumatic spasticity. Neurology, 1984, 15: 526 [↑](#endnote-ref-6)
6. Kamen L, Henney HR, Runyan JD. A practical overview of tizanidine use for spasticity secondary to multiple sclerosis, stroke, and spinal cord injury. Curr Med Res Opin, 2008, 24 (2):425–39 [↑](#endnote-ref-7)
7. Rekand T. Clinical assessment and management of spasticity: a review. Acta Neurol Scand, 2010: 122 (Suppl. 190): 62–66 [↑](#endnote-ref-8)
8. Bajd T, et al. Electrical stimulation in treating spasticity resulting from spinal cord injury. Arch Phys Med Reh, 1985, 66: 526 [↑](#endnote-ref-9)
9. 汪家琮，严尚诚，韩济生等. 穴位经皮神经电刺激（HANS）治疗脊髓损伤引起的痉挛. 中国康复理论与实践，1997，3（3）：111-117 [↑](#endnote-ref-10)
10. Bohannon RW, Smith MB. Inter-rater reliability of a modified Ashworth scale of muscle spasticity. Phys Ther, 1987, 67: 206-207 [↑](#endnote-ref-11)
11. Allison Brashear, Ross Zafonte, Michael Corcoran *et al*. Inter- and Intrarater Reliability of the Ashworth Scale and the Disability Assessment Scale in Patients with Upper-Limb Poststroke Spasticity. Arch Phys Med Rehabil, 2002, 83 (10):1349-54. [↑](#endnote-ref-12)
12. Endicott J, Spitzer RL, Fleiss JL, Cohen J. The global assessment scale: A procedure for measuring overall severity of psychiatric disturbance. Arch Gen Psychiatry. 1976, 33 (6): 766-71. [↑](#endnote-ref-13)
13. Enrique Viosca, José L. Martínez, Pedro L. Almagro, *et al*. Proposal and Validation of a New Functional Ambulation Classification Scale for Clinical Use. Arch Phys Med Rehabil, 2005, 86:1234-8. [↑](#endnote-ref-14)
14. Shah S, Vanclay F, Cooper B. Improving the sensitivity of the Barthel Index for stroke rehabilitation. Journal of Clinical Epidemiology, 1989, 42(8): 703—709 [↑](#endnote-ref-15)
15. Shah S, Vanclay F, Cooper B. Predicting Discharge Status at Commencement of Stroke Rehabilitation.Stroke,1989,20: 766-769 [↑](#endnote-ref-16)
16. Brashear A, Gordon MF, Elovic E, *et al*. Intramuscular injection of botulinum toxin for the treatment of wrist and finger spasticity after a stroke. N Engl J Med, 2002, 347 (6):395–400. [↑](#endnote-ref-17)
